# Evaluating portable EEG: a comparison between two wireless systems (EPOC Flex and LiveAmp) and the wired BrainAmp system

**DOI:** 10.7717/peerj.20416

**Published:** 2026-01-05

**Authors:** Justine Epinat-Duclos, Antoine Rossignon, Jérôme Prado, Jean-Baptiste Van der Henst, Yves Paulignan, Maude Beaudoin-Gobert, Françoise Lecaignard, Nathalie Bedoin

**Affiliations:** 1 Université Claude Bernard Lyon 1, CNRS, INSERM, Centre de Recherche en Neurosciences de Lyon CRNL U1028 UMR5292, PLANETE, Bron, France; 2 Université Claude Bernard Lyon 1, CNRS, INSERM, Centre de Recherche en Neurosciences de Lyon CRNL U1028 UMR5292, EDUWELL, Bron, France; 3 Université Claude Bernard Lyon 1, CNRS, INSERM, Centre de Recherche en Neurosciences de Lyon CRNL U1028 UMR5292, TRAJECTOIRES, Bron, France; 4 Université Claude Bernard Lyon 1, CNRS, INSERM, Centre de Recherche en Neurosciences de Lyon CRNL U1028 UMR5292, CAP, Bron, France; 5 Université Claude Bernard Lyon 1, CNRS, INSERM, Centre de Recherche en Neurosciences de Lyon CRNL U1028 UMR5292, COPHY, Bron, France; 6 Lyon 2 University, Institute of Psychology, Lyon, France

**Keywords:** EEG, ERP, N170, N200, P300, MMN, SSVEP, Alpha power

## Abstract

**Background:**

Recent advances in equipment miniaturization have led to low-cost, portable electroencephalography (EEG) systems that facilitate data collection in real-world settings and with larger samples. Although wireless EEG systems were originally developed for non-research applications, recent studies have provided valuable information to help researchers make informed choices, particularly about participant comfort, mobility during recordings, and data validity. This study aimed to assess the impact of portability by comparing the performance of portable consumer- and research-grade systems (EPOC Saline Flex, EM; LiveAmp, LA) with fixed research-grade systems (BrainAmp, BA).

**Method:**

Continuous EEG was recorded with each system in healthy adults performing five benchmark tasks in fundamental and clinical cognitive neuroscience. Mental states (alpha power variations in open/closed eyes) and unconscious perception (steady-state visual evoked potential, SSVEP) were analyzed through time/frequency methods, while active (N200 and P300 components during active listening and N170 component during face recognition) and passive cognitive processes (Mismatch negativity, MMN component during passive listening) were examined using time/amplitude analyses (event-related potential, ERPs). Our analyses compared system efficiency at native and equalized sampling rates and examined 100%, 75%, and 50% of the datasets to determine the required trial number for satisfactory signal quality.

**Results:**

Despite the smaller amount of signal retained for EM, all systems recorded the expected resting state alpha power decrease and SSVEP responses, with EM showing lower spectral effects ([EM < (LA≈BA)]). ERPs for active (N170, N200, P300) and passive (MMN) processes emerged across all systems, with EM and LA showing lower amplitudes only for N170 compared to BA. Furthermore, the dataset reduction resulted in a decreased N170 at P7 only for EM ([EM < LA < BA]). EM also exhibited shorter latencies for all ERPs except for MMN.

**Conclusion:**

This study provides concrete guidance for designing EEG experiments in real-world settings, with significant potential for investigating children and vulnerable populations. The efficiency of the three EEG systems is more influenced by task duration than sampling rates. A wireless EEG device, such as the EM, can effectively support both time/frequency and time/amplitude analyses in cognitive science, provided that the number of trials is sufficient and latencies are controlled.

## Introduction

The measure of brain activity with electroencephalography (EEG) originated with Hans Berger’s human recordings in 1929. It has since established itself as a popular method among neuroscientists for its non-invasiveness and high temporal resolution. While advances in technology have contributed to improve instrument sensitivity and signal processing, research-grade EEG systems still require extensive wiring. This restricts their use to laboratory settings. Heavy equipment makes it difficult to use EEG for the assessment of naturalistic interactions, sport practice, restless patients, young, as well as for long-recordings or large-scaled studies (Lau-Zhu, Lau & McLoughlin, 2019).

The recent emergence of consumer devices and portable systems has represented a significant advance in EEG research. They are easy to transport, require no application of electrolytic gel and subsequent hair washing, can be easily and quickly installed even outside the laboratory, and allow the participant to move. Therefore, even with a limited budget, EEG data acquisition becomes feasible under ecological conditions (for a review, see Cannard et al., 2020), which encourages the recording of large samples (*e.g*., 505 participants, Kounios et al., 2024), or hitherto little-tested populations, including those at school or at home. The biotech company EMOTIV Systems was among the first to launch consumer EEG devices with the EPOC system. Though the system was initially designed as a hands-free device for video games, it has since found its place in the brain-computer device (BCI) category (Vasiljevic & de Miranda, 2020; Antoniou et al., 2021). This has prompted researchers to evaluate the efficiency of the EPOC system as a research tool. For instance, previous studies concluded that the EMOTIV system can be reliably used in neuroscience research (Sawangjai et al., 2020; Sabio et al., 2024), despite lower signal-to-noise ratio (SNR) of mobile EEG systems than their research-grade counterparts (Radüntz, 2018; Mahdid, Lee & Blain-Moraes, 2020).

In a recent article, Niso et al. (2023) showed the diversity of portable EEG systems on the market and attempted to highlight the characteristics of 48 wireless systems and objectively listed the specific features of each one regarding electrodes, amplifier, and analog-to-digital converter. However, the authors pointed out imprecise information and inconsistences across methodologies in most of the validation studies, suggesting that standardized testing protocols are needed. They also remarked on the lack of direct comparison of portable EEG systems, taking account both portability and signal quality. Limitations are also due to variations in the location and number of electrodes, the sample sizes, and the experimental paradigms used to elicit event-related potentials (ERPs) or changes in neural frequencies. Other authors expressed the need of validation studies including time/frequency signal analyses, and information about the necessary number of trials to obtain expected ERP components (Lau-Zhu, Lau & McLoughlin, 2019). In line with these recommendations, recent efforts have been undertaken to directly compare portable and research-grade EEG systems, but they encountered various obstacles.

For instance, (1) Williams et al. (2020) compared the EMOTIV EPOC with a research-grade system (Neuroscan SynAmps2) using five different paradigms in 20 adults. However, they opted for simultaneous assessment with priority given to EMOTIV positioning at sites of interest—as Neuroscan is widely accepted in research (Badcock et al., 2015)—at the expense of accuracy in electrode location for Neuroscan. This could explain intriguing higher N170 amplitude recorded with EMOTIV than Neuroscan. This limitation could be avoided by an intra-individual sequential assessment of the different EEG systems with a counterbalanced order. (2) Another issue that Ries et al. (2014) faced when comparing the research-grade Biosemi system with the portable Advanced Brain Monitoring (ABM) and EMOTIV systems was the differences in configuration and number of electrodes between the systems. The authors attempted to overcome this limitation by testing a rather large set of participants (*N* = 16) compared to other studies. (3) The validation studies also made it possible to overcome the limitation due to very small numbers of electrodes. For example, Ratti et al. (2017) used intra-individual sequential recordings to compare portable EEG systems: two multichannel medical systems with wet electrodes (B-Alert, Enobio, Barcelona, Spain) and two limited-channel consumer systems with dry electrodes and no impedance control (Muse, Mindwave). The authors found differences in signal quality and greater variability for Muse. However, the signal was only compared from one common site (Fp1) between the four EEG systems. (4) Another limitation in comparing an ambulatory EEG device (OptiEEG, referenced to A1 and A2 positioned at the ear) to a clinical reference device (Natus, referenced to Cz) was differences in the location of the reference electrodes (Shivaraja et al., 2023).

In the present study, we aimed to compare the performance of two portable EEG systems—the EMOTIV EPOC Flex (EM) and the BrainProducts LiveAmp (LA)—to the lab-based BrainProducts BrainAmp system (BA), while addressing many of the shortcoming of previous validation studies highlighted above. The two portable EEG systems were chosen because they were well-suited for the type of developmental cognitive neuroscience research conducted at our research center. Specifically, they are portable and allow for rapid installation because they have wet electrodes (both of which are desirable for testing in schools or at home). Additionally, they have 32 electrodes, which allows for recording of the components that are most studied in our research center and in cognitive psychology and neuroscience generally. Additionally, we were familiar with the BrainAmp system and chose its portative version (*i.e*., LiveAmp) from BrainProduct’s offer. EMOTIV EPOC Flex was selected because of its low cost and the fact that some researchers in our center had already some experience with it. The price difference between the EPOC Flex and the other systems, with a ratio of around 10, prompted us to carry out these investigations in order to measure the advantages and limitations of each system. Among the growing number of validation studies for mobile EEG (for reviews, see Lau-Zhu, Lau & McLoughlin, 2019; Browarska et al., 2021; Sabio et al., 2024), the present one combined a set of conditions that are rarely found together in validation studies: (1) two portable EEG systems have been compared with each other, but also with a reference wired system, which has rarely been done ((but see Khng & Mane, 2020), for a medical-grade system as reference), or with few participants (Ratti et al., 2017); (2) mobile EEG systems were pitted against each other for their efficacy in replicating four expected ERPs, but also two well-established frequency-based (*i.e*., brain oscillations) classical data; (3) all three systems had the same number of electrodes; (4) to compare signals recorded at the same sites, it followed an intra-individual design with fixed order of the tasks, and EEG systems tested in separate sessions with counterbalanced order, instead of simultaneously recording with two systems (Williams et al., 2020); (5) the minimal number of items required to capture the expected changes in brain oscillations and ERPs was assessed by analyzing either 100% or the first 75% and 50% of the data; (6) visual and auditory paradigms were used to provide the scientific community with various and concrete guidelines for implementing experimental protocols with portable EEG systems.

Portable EEG systems were compared here to a lab-based system according to three main dimensions. First, we evaluated whether the three EEG systems could allow for time/frequency analyses of brain activity to infer reliable information about conscious states. Measurable increase in amplitude of alpha power at occipital sites with closed eyes as compared with open eyes (Berger effect) is typically considered to reflect resting state. This spectral feature has already been extracted with mobile EEG systems (Johnstone, Blackman & Bruggemann, 2012; Debener et al., 2015; Cannard, Wahbeh & Delorme, 2021; An et al., 2022), even in children (Bhavnani et al., 2022) and epilepsy patients (Shivaraja et al., 2023). We expected to replicate this effect in healthy adults using our three systems.

Second, we compared the performance of the three systems with measures of steady state visually evoked potential (SSVEP, Regan, 1966). Like the Berger effect, the SSVEP overcomes one of the main weaknesses of conventional ERP experiments, namely the long acquisition time due to a low signal to noise (SNR) (Norcia et al., 2015; Stothart, Smith & Milton, 2020). Participants looked at a red square flashing at three different rates. Analyses focused on the electrophysiological responses in the occipital cortex at the exact frequencies of the displayed stimuli, as already observed for face, word and Arabic numeral processing (Liu-Shuang, Norcia & Rossion, 2014; Lochy, Van Belle & Rossion, 2015; Dzhelyova, Jacques & Rossion, 2016) in both adults and children. A validation study showed comparable SSVEP with the research-grade g.Hlamp and EM systems (Grummett et al., 2015). However, because noise floor was higher with EM, there were concerns about assessing low-frequency spectra (Sabio et al., 2024).

Third, systems were compared based on four ERP components. The first component was the N170, best-known in the domain of face processing (Bentin et al., 1996; Eimer, 2011). Specifically, the N170 was expected to be maximal at occipitotemporal sites in response to facial stimuli compared to aggregates of random features in a discrimination task (Rossion, 2014). The N170 component is relatively easy to observe in children and adults (Taylor, Batty & Itier, 2004), and it has already been recorded with EM (De Lissa et al., 2015; Wehrman et al., 2021). Three other components of interest were also investigated: the N200, the P300, and the mismatch negativity (MMN). To include a perceptual modality other than visual, we chose two auditory paradigms. A Go/Nogo test was used to evaluate variations in N200 and P300 amplitudes and provide indications of *voluntary* inhibition of responses to rare (deviant) phonemes in a stream of frequent (standard) phonemes (Bedoin, Krzonowski & Ferragne, 2013). These stimuli were also used in a passive listening task to assess the MMN, peaking around 200 ms after a deviant onset (Näätänen, 2009) and indexing the *automatic* detection of a unexpected sounds. N200 and P300 have already been successfully recorded using EM, but simultaneously with another system, preventing from comparisons at the same sites (Badcock et al., 2013). Mixed results have been reported for the P300, which was lower when recorded with EM than a medical-grade EEG system, suggesting that numerous trials are necessary (Duvinage et al., 2013). MMN is indicative of phonological development (Linnavalli et al., 2017; Stekić et al., 2023) and it is therefore interesting to know the conditions under which it can be recorded with mobile EEG systems, to favor extensive investigation of speech development in children, phonological deficiencies in patients, or second language learning. Previous studies have indicated a low SNR for the MMN recorded with EM (Badcock et al., 2013) and more trials had to be rejected to record it than with a traditional EEG system (Barham et al., 2017). Therefore, in the present study, analyses of the spectral and ERP recordings conducted on 100%, 75% and 50% of the data could be informative about the minimal number of stimuli enabling researchers to evaluate these various indices of cognitive mechanisms with each of the three investigated EEG systems.

## Methods

Each section closely adheres to the recommendations of the MEEG Committee on Best Practice in Data Analysis and Sharing (COBIDAS) established by the Council of the Organization for Human Brain Mapping (OHBM) (Pernet et al., 2018). The raw data, the output data obtained after processing the EEG files and the scripts used for statistical analyses are available at Zenodo at the link https://zenodo.org/records/15698441.

### Participants

Twenty-one French volunteers, free from neurological or psychiatric conditions and in good health, participated in the study. Data from two participants were excluded due to technical issues and experimenter error, resulting in a final sample of 19 individuals (15 females, four males), aged 19 to 56 years (*M* = 27.6; *SD* = 9.3). All participants provided written informed consent, and the study was approved by the University of Lyon ethics committee (CER-UdL n° 2023-06-15-003).

### General design of the study

The study employed a within-subjects design, divided into three sessions, each featuring a different EEG system. Each participant carried out the three sessions (one or two per day). Each session included five tasks administered in the same order for all EEG systems to minimize variability between sessions: (1) SSVEP, (2) active auditory oddball (standard *vs*. deviant stimuli), (3) passive auditory oddball, (4) face perception, and (5) resting state. Alternating between active and passive tasks aimed at preventing boredom and maintaining participant attention during the approximately 35-min session. In contrast, the order of the EEG systems was counterbalanced to ensure that the three systems were used similarly in the first, second, and third sessions. The total duration of EEG acquisition for one participant, excluding installation time, lasted around 105 min.

### Stimuli

The experiment was conducted in a dedicated sound-attenuated room. Participants were comfortably seated in a chair, with the ventilation system and lights turned off, the curtains drawn, and their phones set to airplane mode throughout the EEG recordings. Stimuli and instructions were delivered using Presentation software (version 23.1) on a laptop with a 15.6″ Full HD (1,920 × 1,080) screen (60 Hz refresh rate). Participants were seated 60 cm from the screen, and auditory stimuli were played through Sennheiser IE 100 Pro Clear in-ear headphones at a comfortable listening level. We used the ERP CORE (Compendium of Open Resources and Experiments) resource material as a starting point to create our variant of the standard ERP paradigms (Kappenman et al., 2021).

#### Resting state paradigm

The task consisted of four 1-min blocks alternating between eyes-open (EO) and eyes-closed (EC) conditions. In the EO condition, participants were shown images of the cosmos. The total duration of the paradigm was 4 min.

#### Steady state visual evoked potential paradigm

The task consisted of six 30-s blocks, each starting with a 2-s white fixation ellipse at the center of the screen (8 × 13 pixels). Stimuli were red squares (200 pixels) on a gray background, flashing at fixed frequencies: 6 Hz (two blocks), 10 Hz (two blocks), or 15 Hz (two blocks). Consequently, squares were displayed for 83.3 ms at 6 Hz, 50 ms at 10 Hz, and 33.3 ms at 15 Hz. The blocks were presented in random order, and participants were instructed to focus on the center of the screen throughout the task, which lasted 3.5 min.

#### Face perception paradigm (N170)

The task consisted of 300 trials, featuring 150 human face images and 150 texture images (400 × 500 pixels) created from scrambled face pixels (from the FACES platform, Ebner, Riediger & Lindenberger, 2010). The faces included young and middle-aged individuals, with equal gender distribution and neutral expressions. Each image was displayed for 300 ms, followed by a 1,000-ms fixation ellipse. Participants pressed a key (Q for face, M for texture) using their left and right index fingers, with key assignments counterbalanced. Two self-paced breaks were provided during the task, which began with a practice sequence of five faces and five textures not included in the main trials. Response times were recorded. The task lasted 7 min.

#### Active auditory oddball paradigm (N200 and P300)

A total of 400 trials were presented, consisting of audio excerpts of the standard /yty/ (80%) and the deviant /ysy/ (20%) pronounced by a female voice. Each trial featured one sound (320 ms) followed by a 1,000-ms interval. Stimuli were pseudo-randomized for each participant, ensuring at least one standard was presented between two deviants. A white fixation ellipse was centered on the screen, and participants were instructed to press the space key for /yty/ and to refrain from pressing for /ysy/. Reaction times for correct responses were recorded. The task was divided into two equal parts with a self-paced break, each beginning with at least four standards, and it lasted 9 min.

#### Passive auditory oddball paradigm (MMN)

Participants were exposed to a sequence of 674 pure tones, each lasting 70 ms. The sequence included 562 tones of 500 Hz (standards, 83.4%) interspersed pseudo-randomly with 112 tones of 550 Hz (deviants, 16.6%) each. Inter-trial interval varied randomly between 550 to 650 ms. The same sequence was used for all participants, with at least two standards between deviants. As in typical settings in passive oddball task (Winkler, 2007), participants watched a silent video throughout the task. By focusing on the movie, the participants engaged attention away from the sounds, which allowed us to measure non-controlled cerebral responses to deviants and standards. There was no break during the MMN task, which lasted 8 min.

### EEG systems

Table 1 presents the characteristics of each system, including channels, electrodes, headset, amplifier resolution, sampling rate, and connectivity.

**Table 1:** Features and comparison of the three EEG systems.

	EPOC Flex	LiveAmp	BrainAmp
	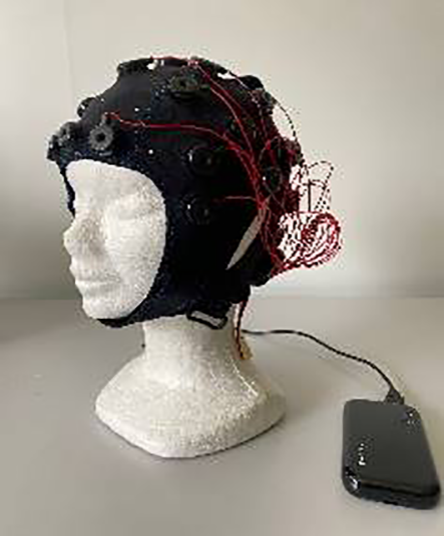	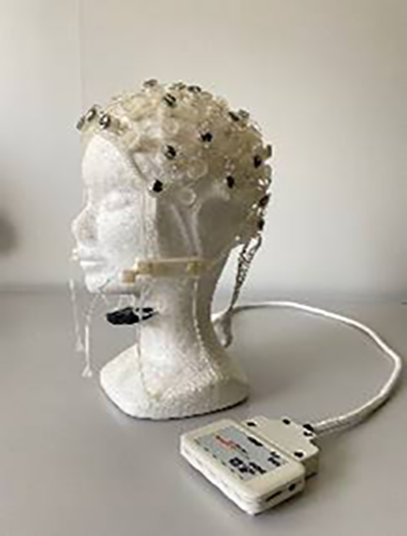	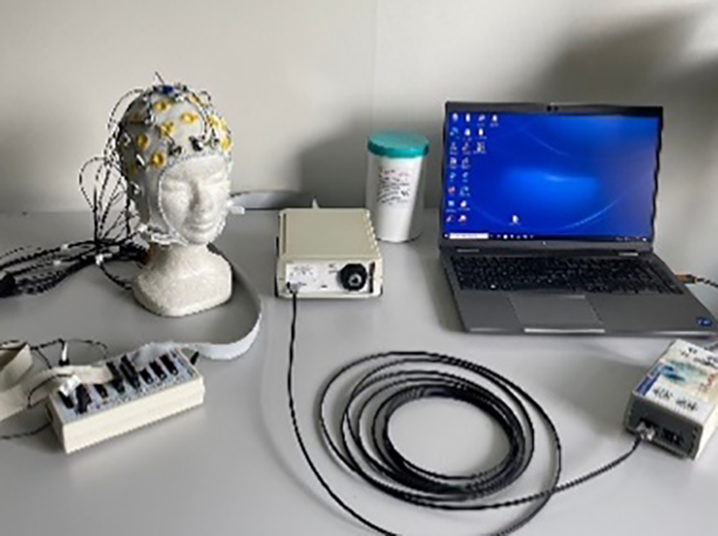
Company	EMOTIV	Brain products	Brain products
Features system	Wireless consumer-grade	Wireless research-grade	Wired research-grade
Channels	32 passive electrodes	32 passive electrodes	32 active electrodes
Ag/AgCl electrodes	Sponge with KCl electrolyte solution	Sponge with NaCl electrolyte solution	Gel-based active electrode
Headset	EasyCap (flexible sensor placement)	R-NET (International 10-10 system)	actiCap slim (International 10-20 system)
Amplifier resolution	14-bit	24-bit	16-bit
Sampling rate	128 Hz	500 Hz	250 Hz
Wireless connectivity	Yes (Bluetooth)	Yes (Wifi)	No
Signal quality check	Yes	Yes	Yes
Impedance check	Yes	Yes	Yes
Setup time (min)	20	20	40
References	Ref: CMS, Gnd: DRL	Ref: FCz, Gnd: Fpz	Ref: FCz, Gnd: AFz

#### LiveAmp & EMOTIV wireless systems

The LiveAmp (LA) research-grade system included a LiveAmp amplifier and a 32-channel R-Net headset (both from Brain Products GmbH). The compact, wireless amplifier communicated with the acquisition computer *via* Wi-Fi, while the R-NET cap used passive wet electrodes arranged according to the international 10-10 system, secured by a flexible silicone structure.

The EM consumer-grade system featured EPOC Flex Saline sensors, a Flex control unit for amplification, an EasyCap cap, and an EMOTIV (EM) Extender. EEG data were wirelessly transmitted *via* Bluetooth from the EPOC Flex control unit to the acquisition computer. The EasyCap system used passive wet electrodes, which were manually positioned according to the international 10-10 system. The EMOTIV Extender received event markers from the laptop presenting the stimuli. EMOTIV products are CE certified and comply with the Radio Equipment Directive (2014/53/EU), but are not intended for medical diagnostics, as stated in EU Directive 93/42/EEC.

#### BrainAmp wired system

The BrainAmp (BA) system included a BrainAmp amplifier, an actiCap cap, and a USB adapter for data transmission and event marker reception. EEG data were acquired using the BrainAmp amplifier with a 32-channel cap featuring active electrodes positioned according to the international 10-20 system and using conductive gel (Supervisc 2). Data were transmitted to the USB adapter *via* optical fiber. Data was sent to the USB adapter through optical fiber. Brain Products GmbH meets the requirements of Annex II (excluding ‘Discussion’) of the Medical Devices Directive 93/42/EEC for neurophysiological research and has implemented a quality assurance system for the design, production, and final inspection of its devices.

### Procedure

#### Electrode positions

**Figure 1:**
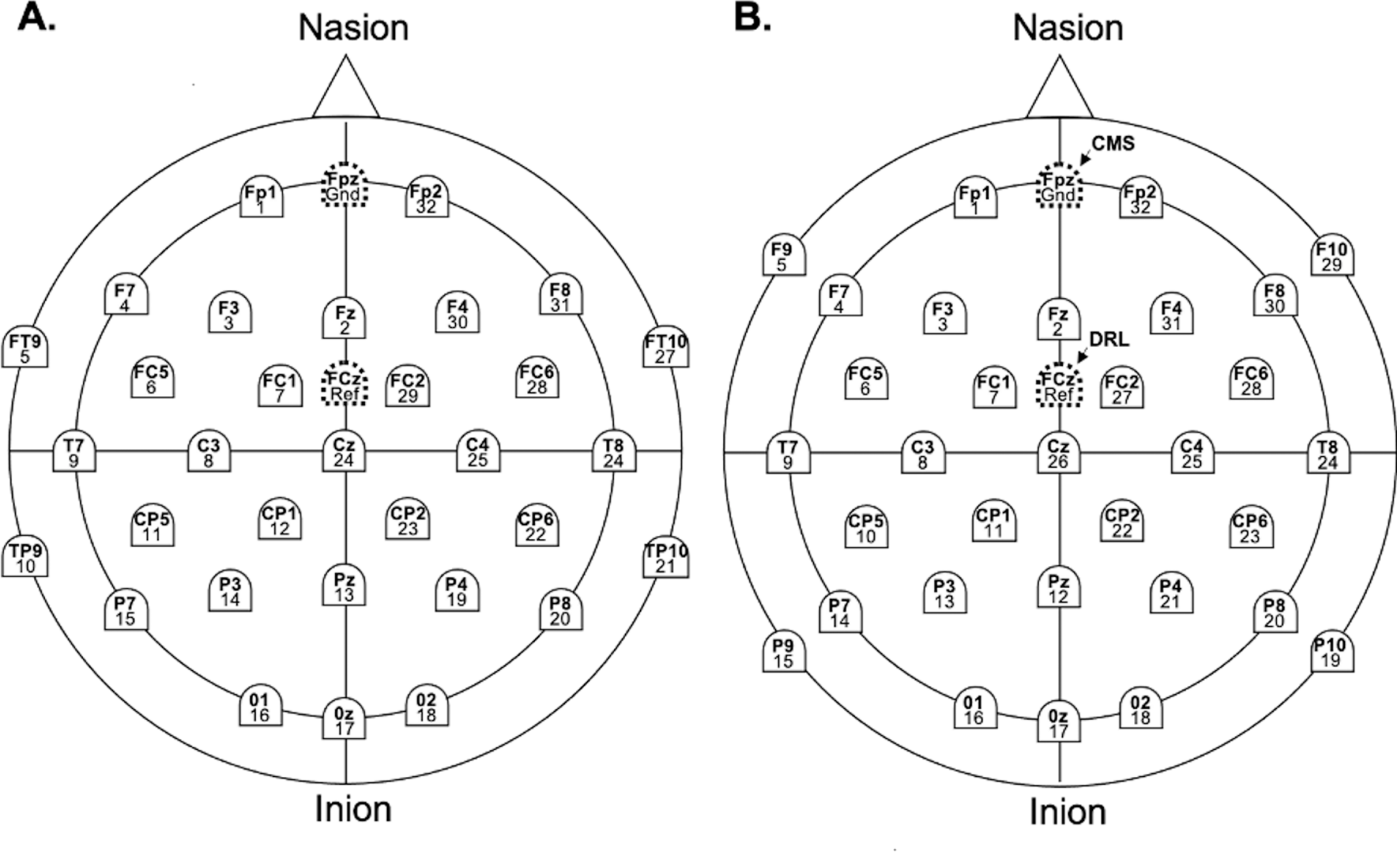
Electrode names and labels for the BA system (A), the LA and EM systems (B).

EEG recordings for each system were acquired on a Windows 11 Pro Dell Precision 3571 laptop, separate from the stimulus presentation computer, using the respective native software (BrainVision Recorder for Brain Products, GmbH and EmotivPRO for EM). Continuous EEG was recorded from 32 scalp sites for the EM and LA systems, with F9, P9, F10, and P10 replaced by FT9, TP9, FT10, and TP10 for the BA system (Fig. 1). In the EM layout, reference electrodes were CMS (Fpz) and DRL (FCz), while LA and BA used Fpz as the ground and FCz as the reference. Impedance was maintained below 50 kΩ for LA and 20 kΩ for BA. Following Williams et al. (2020), Flex electrodes were adjusted until impedance values turned ‘green’, indicating levels below 20 kΩ.

#### Event-marking

Event tagging was performed using a USB-6501 trigger box from NATIONAL INSTRUMENTS-NI, which emitted triggers from the stimulus presentation computer. The triggers were sent *via* a 2.5 mm audio jack to the LiveAmp amplifier for LA and the EMOTIV Extender for EM. As the amplifier had no trigger input channel, the extender box was used to receive the triggers. The extender was connected to the NI board *via* two wires and to the EMOTIV amplifier *via* a USB Mini-A port. For the BA system, triggers were sent to the recording computer *via* a USB adapter. These configurations enabled single-bit event coding, which required an additional step in the processing pipeline to identify all event codes.

#### Offline EEG general processing

Python and the MNE library (Gramfort, 2013; https://doi.org/10.5281/zenodo.592483) were used to match event codes in the EEG recordings with log files from the stimuli presentation software. All subsequent processing steps were conducted using BrainVision Analyzer software (version 2.2).

#### System sampling rate and dataset

BA and LA data were downsampled to 128 Hz to match the EM’s native sampling rate, referred to as Pack-100a. For the three ERP tasks, an additional analysis retained LA and BA recordings at their native rates of 500 and 250 Hz, respectively (Pack-100b). Further analyses on raw data included the first 75% or 50% of trials for each task (Pack-75, Pack-50).

#### Common steps for signal processing

The EEG signal was filtered using a bandpass filter (0.016 to 30 Hz for Resting State, Active Auditory, and Passive Auditory tasks; 0.01 to 55 Hz for SSVEP; 0.1 to 40 Hz for Face Perception) and a 50 Hz notch filter. The data was then re-referenced to the common average.

#### Resting state processing

Continuous EEG was segmented into two 60-second segments for the eyes-closed (EC) and eyes-open (EO) conditions. A wavelet transform using Morlet complex waveforms (1 to 40 Hz with Gabor normalization) was applied to these segments. Spectral power from the two segments in each condition was averaged afterwards, and alpha band power (8.5–12.7 Hz) was extracted for each participant and system at the O1, Oz, and O2 electrodes before final averaging.

#### SSVEP processing

Continuous EEG was segmented for 6, 10 and 15 Hz conditions, resulting in two 30-s segments for each one, which have been finally averaged for each condition at O1, Oz and O2. Spectral power over these electrodes was calculated with a Fast Fourier Transform (0.05 Hz resolution using a Hanning window). For each frequency bin, a SNR was calculated by dividing the amplitude of a frequency bin by the average of the 20 surrounding bins (10 on each side, excluding the two immediate neighbors).

#### ERP processing

For ERPs, artifacts corresponding to deflection greater than 500 µV over 800 ms were marked as large muscular events in the raw data for each electrode. Electrodes with artifacts longer than 5% of the total recording time were deemed faulty and removed. No interpolation was performed. The data was epoched from −200 to 800 ms relative to stimulus onset, except for the passive auditory task, where epochs were from −50 to 560 ms due to variable inter-stimulus intervals (ISI) that could cause overlap.

An independent component analysis (ICA) was conducted and components that were manually identified as blinks or saccade artifacts were corrected. After data had been epoched, a second automatic artifact rejection excluded epochs with deflections exceeding 200 µV. No other noise reduction step was conducted afterwards.

#### N170

One subject was excluded from all systems due to a high rejection rate (94.3%) in the EM recordings. For baseline correction, the mean voltage of the 200-ms prestimulus interval was subtracted from each epoch. Face and texture epochs were averaged separately for each participant across the three systems.

The N170 peak was identified at P7 and P8 electrodes as the most negative voltage between 100 and 160 ms, with temporal windows set to 120–160 ms for LA and BA and 100–140 ms for EM. The P100 peak was determined as the maximum positive voltage between 60 and 120 ms at P7 and P8, with windows of 80–120 ms for LA and BA and 60–100 ms for EM. In the face condition, the N170 peak latency and the peak-to-peak (P2P) amplitude difference between P100 and N170 were extracted for each participant and system.

#### N200 and P300

The mean voltage from the 200-ms prestimulus interval was subtracted from each epoch. ERPs time-locked to stimulus onset were averaged separately for standards and deviants at every electrode, system, and participant. Standards immediately following a deviant were excluded (Bedoin, Krzonowski & Ferragne, 2013). The average waveforms for standards were then subtracted from those for deviants to extract ERP responses for each participant. N200 intervals were determined from the grand average ERP at Fz, peaking around 270 ms for BA and LA, and 260 ms for EM, with temporal windows set to 200–280 ms for BA, LA and EM. The P300 peak was identified at around 380 ms for BA, LA and EM, using a time window of 280–420 ms for all systems. N200 and P300 peak amplitude and latency were analyzed at Fz, Cz, and Pz. One participant was excluded from the analysis for all systems due to excessive faulty electrodes at Fz, Pz, and 11 adjacent electrodes during EM processing.

#### MMN

Frequencies below 0.5 Hz and above 20 Hz were filtered out, and baseline correction was performed by subtracting the mean voltage from the 50 ms prestimulus interval. ERPs time-locked to sound onset were averaged for deviants and standards preceding a deviant to compute the MMN as the difference signal (deviant waveform minus standard waveform). Visual inspection of the average difference waveform at Fz indicated that the MMN peaked around 190 ms for LA, 200 ms for BA and 140 ms for EM, leading to the selection of a 100–250 ms time window. The area under the curve (AUC) has been chosen as the amplitude measure for the MMN (Pekkonen et al., 1993). It was computed following Beauchemin & De Beaumont (2005) guidelines over a 50 ms window centered on the MMN peak in the grand average ERP. Statistical analyses were conducted on peak amplitude, peak latency, and AUC across Fz, Cz, and Pz, with time windows adjusted for each system and pack (Table 2). One participant was excluded from all analyses due to numerous noisy electrodes at Fz, Pz, and 11 adjacent sites during the processing steps of the EM recording.

**Table 2:** Mean MMN peak latency across all analysis and time windows for area calculation.

	MMN peak latency (ms)			Center of the temporal window for area calculation (ms)	Temporal window (ms)	
Pack	System	Fz	Cz		Start	End
100a	EM	141	188	164.5	139.5	189.5
	LA	203	211	207	182	232
	BA	195	188	191.5	166.5	216.5
100b	EM	141	188	164.5	139.5	189.5
	LA	190	188	189	164	214
	BA	200	212	206	181	231
75b	EM	141	188	164.5	139.5	189.5
	LA	190	190	190	165	215
	BA	200	212	206	181	231
50b	EM	141	180	160.5	135.5	185.5
	LA	190	208	199	174	224
	BA	200	200	200	175	225

## Data analyses

Statistical analyses were conducted using Python with the *pingouin* and *scipy.stats* libraries. Expected phenomena (alpha power, harmonic responses, ERPs) were assessed with two-tailed one-sample *t*-tests against zero. Repeated-measures ANOVAs were performed, followed by two-tailed bilateral *t*-tests with Bonferroni adjustment. Effect sizes were reported for ANOVAs (partial eta-squared, η^2^; small = 0.01, medium = 0.06, large = 0.14) and *t*-tests (Cohen’s *d*; small = 0.2, medium = 0.5, large = 0.8), with all results expressed as absolute values.

### Resting state

The difference alpha power between EO and EC conditions was computed for each participant and compared to zero using a two-tailed *t*-test. An ANOVA was conducted on this difference with system (BA, EM, LA) as within-subject factor.

### SSVEP

The SNR at the fundamental components and their first two harmonics (12, 18 Hz for the 6 Hz condition; 20, 30 Hz for the 10 Hz condition; 30, 45 Hz for the 15 Hz condition) were averaged. The presence of harmonic responses was assessed using *t*-tests against zero for the pooled SNR values. A two-way ANOVA was performed on the SNR using system (EM, LA, BA) and condition (6, 10, 15 Hz) as within-subject factors.

### N170

The P100-N170 difference in peak amplitude was compared to zero by a *t*-test at P7 and P8. ANOVAs using the EEG system (EM, LA, BA) as within-subject factor were conducted on N170 peak latency and P100-N170 peak amplitude difference, at P7 and P8.

### N200 and P300

The presence of significant N200 and P300 waves was assessed using *t*-tests against zero for peak amplitude at Fz, Cz and Pz. ANOVAs were performed on N200 and P300 latencies and amplitudes at Fz, Cz and Pz with EEG system as within-subject factor (three levels).

### MMN

Significant MMN was assessed with one-tailed *t*-tests comparing the AUC and mean peak amplitude to zero at Fz, Cz and Pz. ANOVAs were performed on latency, amplitude, and AUC of the MMN at these electrode sites using EEG system (three levels) as within-subject factor.

### Supplementary analyses

We conducted a series of additional analyses to further assess the reliability and precision of the ERP measures obtained across the three EEG systems. First, Pearson correlations were calculated to assess subject-by-subject measurement consistency across the three systems. Second, we computed the standardized measurement error (SME) for each ERP component, following the procedure described by Luck et al. (2021). The SME provides an estimate of the trial-level variability in amplitude and latency measures, and thus complements the correlation analysis by quantifying measurement precision at the individual level. The SME for each system was compared with the benchmark from Zhang & Luck (2023) (when available at the same electrode sites) using Mann-Whitney tests. The systems were also pitted against each other using Friedman tests.

## Results

### Signal quality

As can be seen in Table 3, the EM system yielded less exploitable data than LA and BA systems. The EM headset had to be changed in the middle of a session for two participants due to a lost connection between the amplifier and the acquisition computer. More triggers were lost and more electrodes were declared faulty within the region of interest with EM than the other systems, leading to exclude one participant from the analysis of N200, P300 and MMN for every system. Another participant has been excluded for N170 for every system, because of a high percentage of rejected epochs with EM system.

**Table 3:** ERP signal quality of EM, LA and BA systems. Standard errors are in brackets.

EEG system	Number of lost triggers, mean (SE) [range]	Number of faulty electrodes, mean (SE) [range]	Average percentage of rejected epochs, mean % (SE) [range]		
			N170	N200 & P300	MMN
EM	4.10 (1.30) [0–19]	1.80 (0.70) [0–13]	29.00 (4.8) [2.3–94.3]	2.40 (0.6) [0–11.0]	17.70 (0.50) [15.3–22.7]
LA	0.05 (0.05) [0–1]	0.05 (0.05) [0–1]	0.50 (0.2) [0.0–2.7]	0.40 (0.1) [0.0–2.5]	0.30 (0.10) [0.0–1.3]
BA	0.00 (0.00) [0–0]	0.0 (0.00) [0–0]	0.90 (0.3) [0.0–4.7]	0.20 (0.1) [0.0–1.3]	0.05 (0.03) [0.0–0.3]

### Resting state results

When compared to zero, the difference in alpha power between the EO and EC conditions (Berger Effect) was significant and large-sized for each EEG system whether calculated in Pack-100, 75, or 50 (Cohen’s *d*_s_ > 1.03, Table 4, left).

**Table 4:** Mean difference in alpha power for each pack and each system as compared to zero (left panel), and between-system comparisons for the Berger Effect (right panel).

			Comparison to zero			Between systems comparison		
Pack	EEG system	Mean difference (*SE*)	*t*-value (*df* = 18)	*p-*value	Cohen’s *d*	*t*-value (*df* = 18)	*p-*value	Cohen’s *d*
100	EM	1.93 (0.43)	4.49	<0.001***	1.03			
	LA	3.31 (0.42)	7.79	<0.001***	1.79			
	BA	3.81 (0.71)	5.39	<0.001***	1.24			
	EM/LA					3.23	0.014*	0.74
	EM/BA					2.87	0.030*	0.74
	LA/BA					1.11	0.842	0.19
75	EM	2.00 (0.43)	4.64	<0.001***	1.06			
	LA	3.41 (0.44)	7.83	<0.001***	1.80			
	BA	3.86 (0.75)	5.14	<0.001***	1.18			
	EM/LA					3.24	0.014*	0.75
	EM/BA					2.79	0.037*	0.70
	LA/BA					0.97	0.999	0.17
50	EM	2.12 (0.49)	5.01	<0.001***	1.15			
	LA	3.49 (0.80)	7.65	<0.001***	1.76			
	BA	4.00 (0.92)	5.22	<0.001***	1.20			
	EM/LA					3.15	0.016*	0.72
	EM/BA					2.67	0.047*	0.70
	LA/BA					1.11	0.844	0.18

**Note:**

* *p* ≤ 0.05.

*** *p* ≤ 0.001.

The significant main effect of system was large in size for Pack-100 (*F*(2, 36) = 7.04, *p* = 0.007, η_p_^2^ = 0.28), Pack-75 (*F*(2, 36) = 6.62, *p* = 0.008, η_p_^2^ = 0.27), and Pack-50 (*F*(2, 36) = 6.36, *p* = 0.011, η_p_^2^ = 0.26), reflecting a lower Berger Effect with EM than LA and BA (Table 4, right).

### SSVEP results

**Figure 2:**
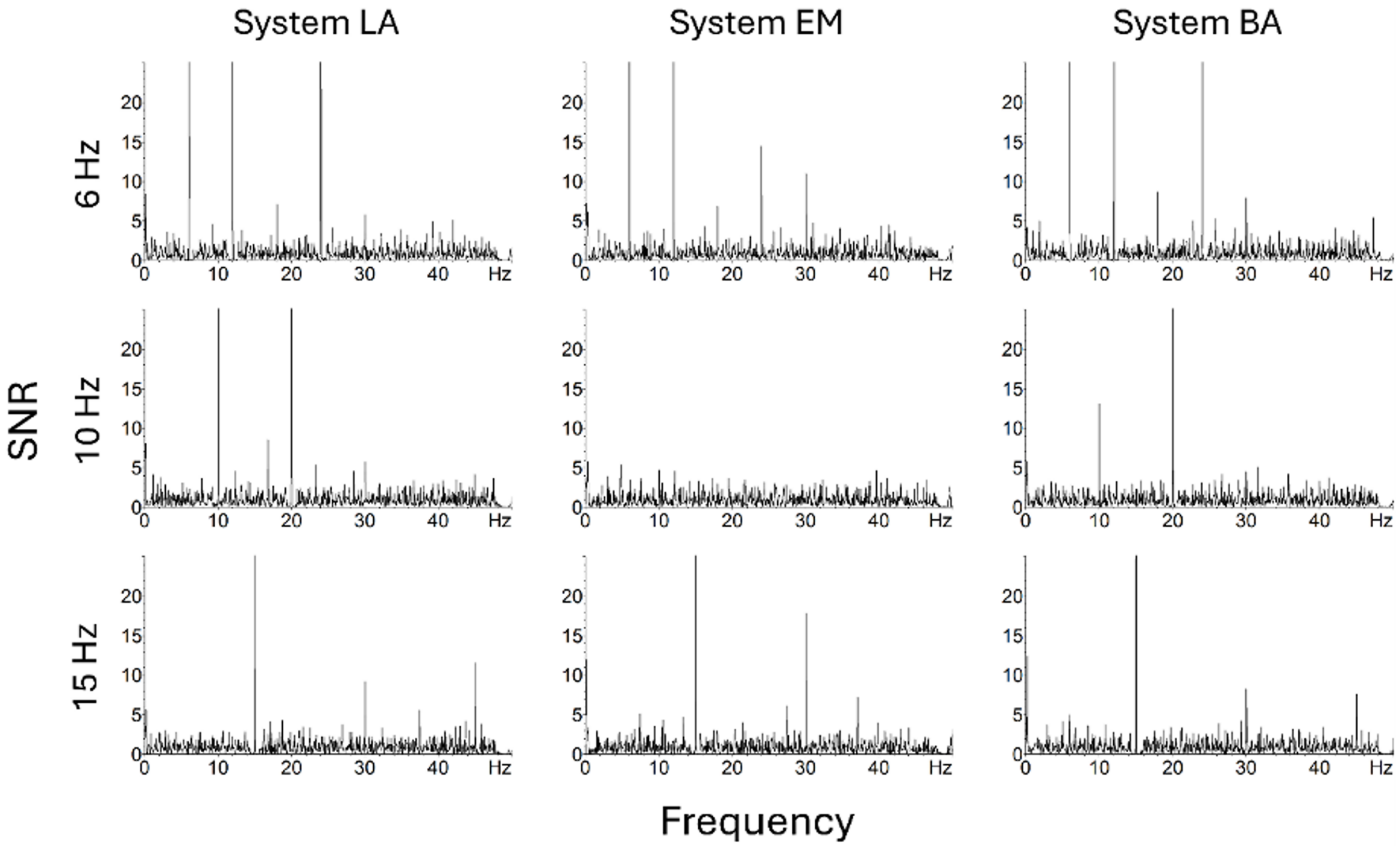
Amplitude spectra expressed as SNR across occipital electrodes (O1, Oz, O2) for the three systems and three conditions (fundamental component = 6, 10 or 15 Hz) in Pack-100a.

The SNR was significantly higher than zero in the 6, 10, and 15 Hz conditions for every system (Fig. 2, Table 5), and the effect sizes were large (*d*_s_ > 1.25) for every system and Pack.

**Table 5:** Comparisons of the SNR value to zero, for each pack, system, and condition.

Pack	EEG system	Condition	SNR (*SE*)	*t*-value (*df* = 36)	*p*-value	Cohen’s *d*
100	EM	6 Hz	13.07 (3.40)	3.85	<0.001***	1.25
		10 Hz	10.81 (2.44)	4.44	<0.001***	1.51
		15 Hz	14.91 (3.00)	4.98	<0.001***	1.60
	LA	6 Hz	21.51 (4.62)	4.66	<0.001***	1.44
		10 Hz	18.88 (3.03)	6.23	<0.001***	2.02
		15 Hz	31.56 (3.62)	8.73	<0.001***	1.53
	BA	6 Hz	29.59 (6.02)	4.92	<0.001***	1.61
		10 Hz	26.81 (6.15)	4.73	<0.001***	2.83
		15 Hz	38.23 (8.77)	5.83	<0.001***	1.89
75	EM	6 Hz	10.28 (2.65)	3.87	<0.001***	1.26
		10 Hz	10.11 (2.43)	4.16	<0.001***	1.35
		15 Hz	13.47 (2.81)	4.79	<0.001***	1.55
	LA	6 Hz	17.72 (4.15)	4.27	<0.001***	1.38
		10 Hz	16.63 (2.65)	6.27	<0.001***	2.03
		15 Hz	26.39 (2.92)	9.03	<0.001***	2.93
	BA	6 Hz	24.58 (5.18)	4.74	<0.001***	1.54
		10 Hz	23.77 (5.30)	4.48	<0.001***	1.45
		15 Hz	32.96 (5.93)	5.56	<0.001***	1.80
50	EM	6 Hz	7.28 (1.82)	4.00	<0.001***	1.30
		10 Hz	7.06 (1.64)	4.29	<0.001***	1.39
		15 Hz	10.03 (1.90)	5.29	<0.001***	1.72
	LA	6 Hz	12.99 (2.71)	4.79	<0.001***	1.55
		10 Hz	12.12 (1.99)	6.09	<0.001***	1.98
		15 Hz	17.70 (1.83)	9.70	<0.001***	3.15
	BA	6 Hz	16.80 (3.56)	4.72	<0.001***	1.53
		10 Hz	17.76 (4.19)	4.24	<0.001***	1.37
		15 Hz	22.31 (3.85)	5.79	<0.001***	1.88

**Notes:**

*** *p* ≤ 0.001.

In Pack-100, the significant main effect of system (*F*(2, 36) = 23.93, *p* < 0.001, η_p_^2^ = 0.57) was due to lower SNR with EM than LA (*t*(18) = 8.17, *p* < 0.001, *d* = 0.97) and BA (*t*(18) = 5.45, *p* < 0.001, *d* = 1.11). There was no main effect of condition neither Condition × System interaction. However, the SNR was very low with EM at 10 Hz condition for the fundamental component and its harmonics (Fig. 2).

In Pack-75, there was only a significant main effect of system (*F*(2, 36) = 18.15, *p* < 0.001, η_p_^2^ = 0.50), reflecting lower SNR with EM than LA (*t*(18) = 6.71, *p* < 0.001, *d* = 0.93) and BA (*t*(18) = 4.94, *p* < 0.001, *d* = 1.05). Similarly, in Pack-50, the main effect of system (*F*(2, 36) = 16.44, *p* < 0.001, η_p_^2^ = 0.48) reflected lower SNR with EM than LA (*t*(18) = 6.53, *p* < 0.001, *d* = 0.98) and BA (*t*(18) = 4.77, *p* < 0.001, *d* = 1.03).

### N170 results

To assess the absence of any bias due to the EEG system on overall response speed, mean response times and SD were submitted to Wilcoxon tests, corrected for multiple comparisons. No significant difference was observed neither for response speed (EM-LA, *V* = 82, *p* = 0.615; EM-BA, *V* = 111, *p* = 0.841; LA-BA, *V* = 107, *p* = 0.956) nor speed variability (EM-LA, *V* = 108, *p* = 0.927; EM-BA, *V* = 139, *p* = 0.216; LA-BA, *V* = 145, *p* = 0.143).

In **face condition** and Pack-100 with native sampling rate (Pack-100a for EM, Pack-100b for LA and BA), the P2P **amplitude** difference (P100-N170, hereafter, N170) significantly differed from zero at P7 and P8, with large effect sizes for every system (1.22 < all *d*_s_ < 2.06) (Table 6). N170 remained large-sized in Packs-75 and 50 for the three systems (1.12 < all *d*_s_ < 2.00), and with lower sampling rate (Pack-100a) for LA and BA (1.22 < all *d*_s_ < 1.93).

**Table 6:** Peak latency of the N170 for face condition, and comparisons of the Peak-to-Peak (P2P = P100-N170) amplitude difference to zero, for each pack, sampling rate, system, and electrode site.

Data set		EEG System	Site	N170 peak latency, ms (SE)	P2P, mV (SE)	*t*-value (*df* = 17)	*p*-value	Cohen’s *d*	Between system amplitude comparison		
Pack	Sampling								*t* (*df* = 17)	*p-*value	*d*
100a	128 Hz	EM	P7	119.49 (2.51)	5.00 (0.96)	5.20	<0.001***	1.23	–	–	–
			P8	121.53 (2.62)	4.11 (0.79)	5.22	<0.001***	1.23	–	–	–
		LA	P7	141.50 (2.75)	5.66 (1.02)	5.57	<0.001***	1.31	–	–	–
			P8	140.19 (3.43)	6.43 (0.89)	7.22	<0.001***	1.70	–	–	–
		BA	P7	140.19 (2.64)	8.29 (1.01)	8.18	<0.001***	1.93	–	–	–
			P8	139.76 (2.89)	10.14 (1.45)	6.97	<0.001***	1.64	–	–	–
		EM-LA	P7	–	–	–	–	–	0.96	0.999	0.16
		EM-BA	P7	–	–	–	–	–	4.55	<0.001***	0.79
		LA-BA	P7	–	–	–	–	–	3.86	0.004**	0.61
		EM-LA	P8	–	–	–	–	–	2.44	0.077	0.65
		EM-BA	P8	–	–	–	–	–	4.37	0.001**	1.21
		LA-BA	P8	–	–	–	–	–	3.17	0.017*	0.73
100b	500 Hz	LA	P7	141.56 (3.16)	5.86 (1.02)	5.74	<0.001***	1.35	–	–	–
			P8	139.78 (3.39)	6.62 (0.91)	7.30	<0.001***	1.72	–	–	–
	250 Hz	BA	P7	141.33 (2.78)	8.65 (0.99)	8.74	<0.001***	2.06	–	–	–
			P8	139.33 (2.77)	10.51 (1.49)	7.07	<0.001***	1.67	–	–	–
		EM-LA	P7	–	–	–	–	–	1.19	0.754	0.21
		EM-BA	P7	–	–	–	–	–	5.17	<0.001***	0.88
		LA-BA	P7	–	–	–	–	–	4.18	0.002**	0.65
		EM-LA	P8	–	–	–	–	–	2.62	0.054	0.70
		EM-BA	P8	–	–	–	–	–	4.63	<0.001***	1.27
		LA-BA	P8	–	–	–	–	–	3.22	0.015*	0.74
75	128 Hz	EM	P7	120.86 (2.45)	5.15 (0.96)	5.36	<0.001***	1.26	–	–	–
			P8	121.53 (2.54)	4.12 (0.83)	4.97	<0.001***	1.17	–	–	–
	500 Hz	LA	P7	143.44 (3.50)	5.94 (1.03)	5.77	<0.001***	1.36	–	–	–
			P8	142.22 (3.92)	6.70 (0.90)	7.48	<0.001***	1.76	–	–	–
	250 Hz	BA	P7	141.78 (2.73)	8.75 (1.03)	8.48	<0.001***	2.00	–	–	–
			P8	141.11 (3.42)	10.44 (1.52)	6.85	<0.001***	1.61	–	–	–
		EM-LA	P7	–	–	–	–	–	1.05	0.926	0.19
		EM-BA	P7	–	–	–	–	–	4.79	<0.001***	0.85
		LA-BA	P7	–	–	–	–	–	4.05	0.003**	0.64
		EM-LA	P8	–	–	–	–	–	2.62	0.053	0.70
		EM-BA	P8	–	–	–	–	–	4.46	0.001**	1.21
		LA-BA	P8	–	–	–	–	–	3.11	0.019*	0.70
50	128 Hz	EM	P7	121.32 (2.87)	5.32 (1.04)	5.09	<0.001***	1.20	–	–	–
			P8	121.09 (2.47)	4.11 (0.86)	4.77	<0.001***	1.12	–	–	–
	500 Hz	LA	P7	140.89 (3.36)	6.09 (1.04)	5.88	<0.001***	1.39	–	–	–
			P8	142.56 (3.87)	6.98 (0.90)	7.72	<0.001***	1.82	–	–	–
	250 Hz	BA	P7	141.33 (2.71)	9.10 (1.10)	8.24	<0.001***	1.94	–	–	–
			P8	140 (2.84)	10.53 (1.52)	6.95	<0.001***	1.64	–	–	–
		EM-LA	P7	–	–	–	–	–	0.98	0.999	0.18
		EM-BA	P7	–	–	–	–	–	4.86	<0.001***	0.83
		LA-BA	P7	–	–	–	–	–	4.27	0.002**	0.66
		EM-LA	P8	–	–	–	–	–	2.79	0.038*	0.76
		EM-BA	P8	–	–	–	–	–	4.60	<0.001***	1.23
		LA-BA	P8	–	–	–	–	–	2.94	0.027*	0.67

**Notes:**

* *p* ≤ 0.05.

** *p* ≤ 0.01.

*** *p* ≤ 0.001.

**Figure 3:**
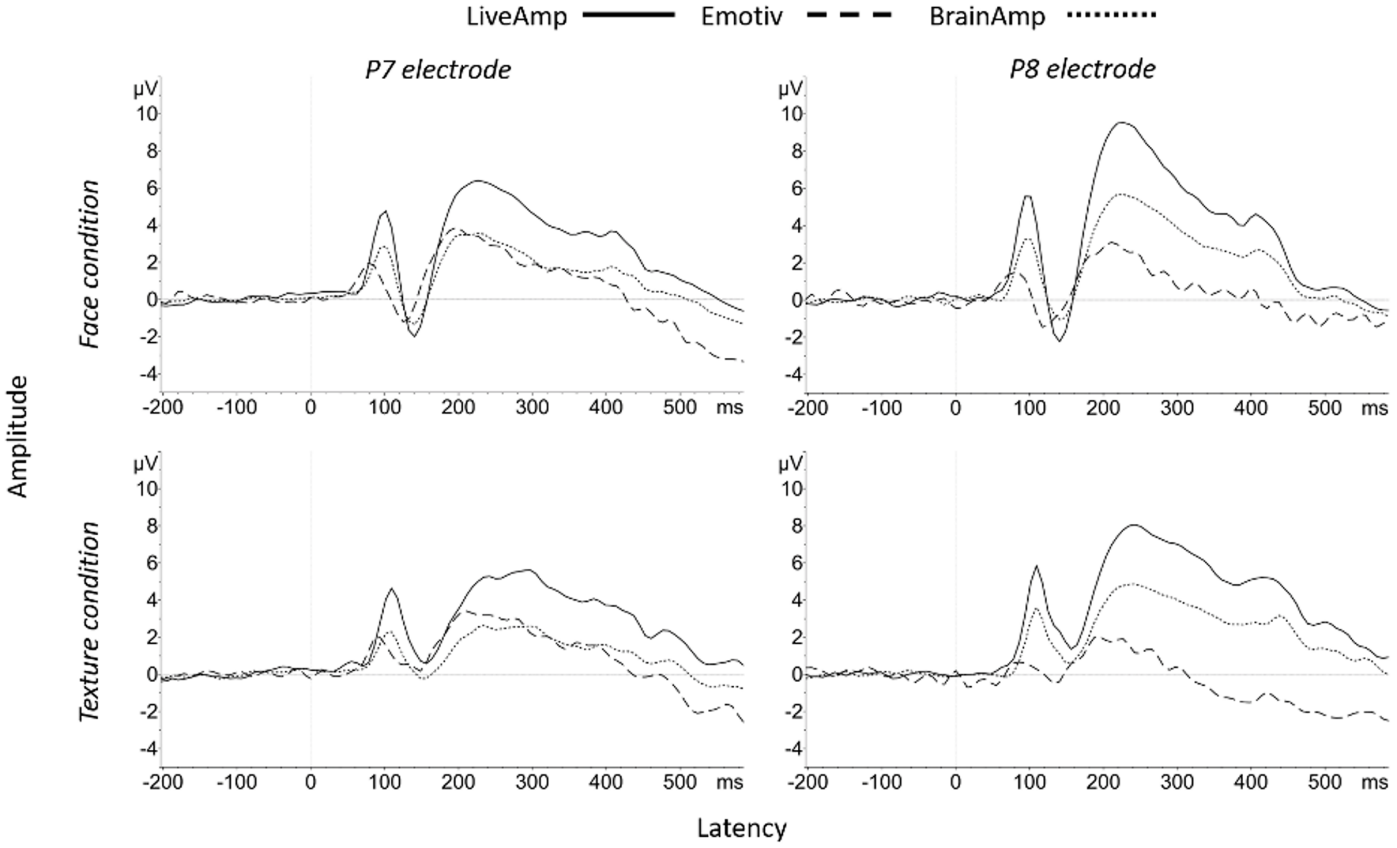
Group N170 ERP waveforms for the three systems at P7 and P8 electrode sites in response to face (top) and texture (bottom).

For Pack-100 and native sampling rate, the significant main effect of system on P2P amplitude at P7 (*F*(2, 34) = 14.81, *p* < 0.001, η_p_^2^ = 0.47) and P8 (*F*(2,34) = 14.57, *p* < 0.001, η_p_^2^ = 0.46) reflected higher N170 for BA than EM and LA, but no EM-LA difference (Fig. 3, Table 6). This pattern of results was confirmed by the main effect of system with a lower sampling rate (Pack-100a: P7, *F*(2, 34) = 12.36, *p* < 0.001, η_p_^2^ = 0.42; P8, *F*(2, 34) = 13.29, *p* < 0.001, η_p_^2^ = 0.44), and Pack-75 (P7, *F*(2, 34) = 13.30, *p* < 0.001, η_p_^2^ = 0.44; P8, *F*(2, 34) = 13.73, *p* < 0.001, η_p_^2^ = 0.45). For Pack-50, the significant main effect of system at P7 (*F*(2, 34) = 13.82, *p* < 0.001, η_p_^2^ = 0.45) reflected higher N170 for BA than EM and LA, but at P8 (*F*(2, 34) = 13.89, *p* < 0.001, η_p_^2^ = 0.45) it reflected higher N170 with BA than LA and higher N170 with LA than EM (Table 6).

Regarding N170 **latency** for face condition, the significant main effect of system in Pack-100 at native sampling rate (P7, *F*(2, 34) = 76.08, *p* < 0.001, η_p_^2^ = 0.82; P8, *F*(2, 34) = 71.62, *p* < 0.001, η_p_^2^ = 0.81) reflected shorter latencies for EM than LA and BA (Table 6, Fig. 3). This pattern remained in Pack-100a, according to the main system effect (P7, *F*(2, 34) = 77.45, *p* < 0.001, η_p_^2^ = 0.82; P8, *F*(2, 34) = 66.64, *p* < 0.001, η_p_^2^ = 0.80), as well as in Pack-75 (P7, *F*(2, 34) = 60.14, *p* < 0.001, η_p_^2^ = 0.78; P8, *F*(2, 34) = 52.32, *p* < 0.001, η_p_^2^ = 0.75), and Pack-50 (P7, *F*(2, 34) = 41.64, *p* < 0.001, η_p_^2^ = 0.71; P8, *F*(2, 34) = 39.34, *p* < 0.001, η_p_^2^ = 0.70).

**Figure 4:**
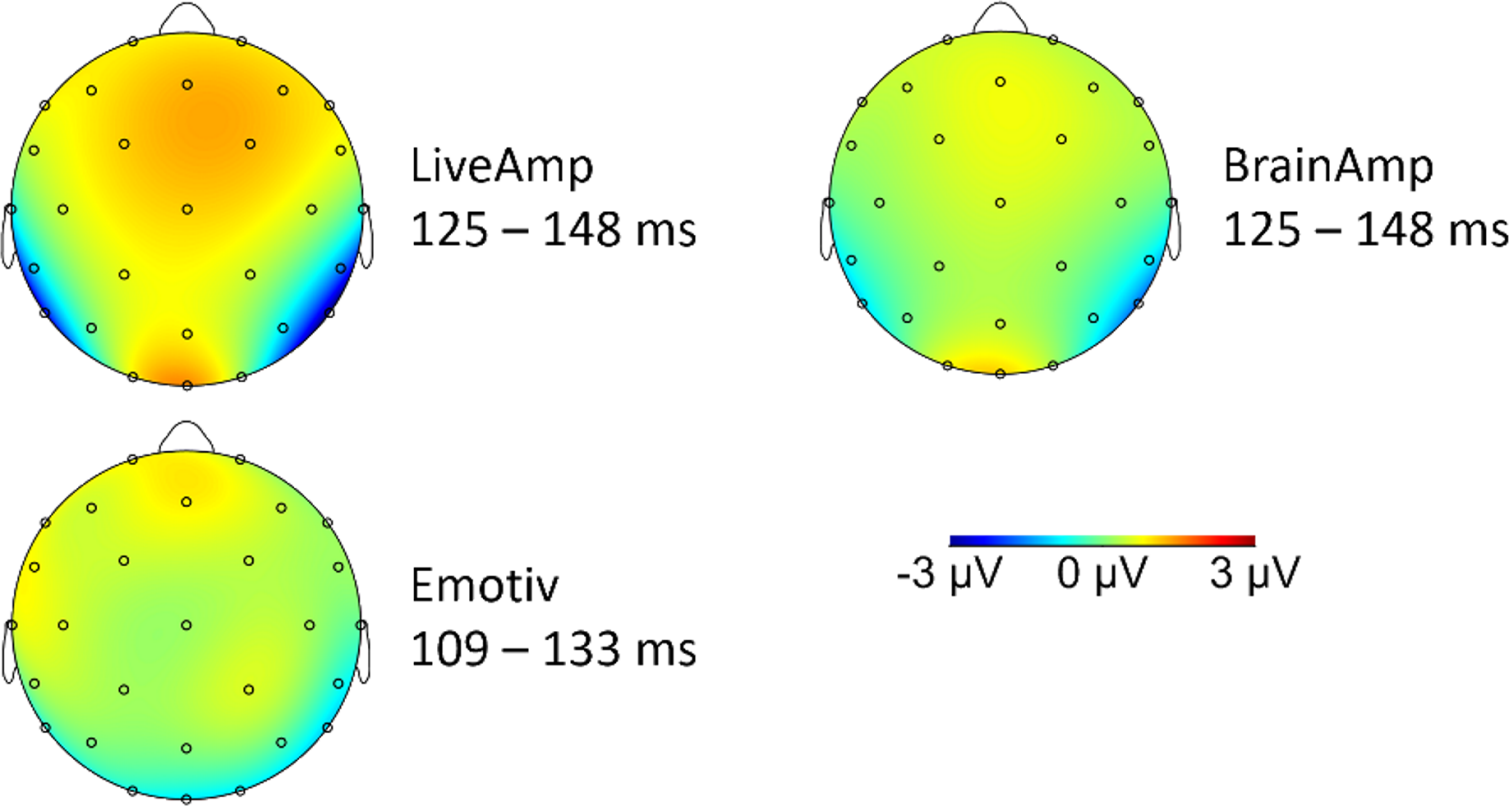
Topographic distribution of the signal for the difference between face and texture conditions.

The typical parietal N170 saliency for faces was observed with LA and B—less with EM—, at least when the face-texture difference was represented (Fig. 4).

In **texture condition**, N170 for Pack-100a significantly exceeded zero at P7 and P8 (Table 7), and the effect size was large for LA and BA, and medium for EM. The examination of Fig. 3 suggested the classical decrease in amplitude and increase in latency of the N170 at posterior electrodes in texture condition as compared with face condition (Rossion, 2014), especially for LA and BA.

**Table 7:** Peak latency of the N170 for texture condition, and comparisons of the Peak-to-Peak (P2P = P100-N170) amplitude difference to zero, for each system and electrode site, with equated sampling rates (Pack-100a).

Data set		EEG system	Site	N170 peak latency, ms (SE)	P2P, mV (SE)	*t*-value (*df* = 17) against zero	*p*-value
Pack	Sampling						
100a	128 Hz	EM	P7	122.70 (0.18)	2.83 (0.10)	3.65	0.002**
			P8	119.36 (0.19)	1.76 (0.08)	3.27	0.005**
		LA	P7	141.93 (0.18)	2.94 (0.09)	4.33	<0.001***
			P8	139.32 (0.18)	3.55 (1.10)	4.78	<0.001***
		BA	P7	141.93 (0.18)	4.09 (0.11)	4.62	<0.001***
			P8	138.89 (0.19)	4.71 (0.12)	4.22	<0.001***

		Latency comparisons			Amplitude comparisons		
		*T* *t* (*df* = 17)	*p-*value	*d*	*t* (*df* = 17)	*p-*value	*d*
EM-LA	P7	6.29	<0.001***	1.85	0.22	0.999	0.04
EM-BA	P7	6.15	<0.001***	1.79	2.21	0.125	0.36
LA-BA	P7	0.00	0.999	0.00	2.21	0.124	0.34
EM-LA	P8	6.06	<0.001***	1.83	4.52	<0.001***	0.65
EM-BA	P8	5.36	<0.001***	1.69	3.49	0.008**	0.79
LA-BA	P8	0.32	0.999	0.04	1.37	0.562	0.29

**Notes:**

** *p* ≤ 0.01.

*** *p* ≤ 0.001.

The main effect of system for Pack-100a was significant at P8 (*F*(2, 34) = 8.38, *p* < 0.001, η_p_^2^ = 0.33), reflecting lower N170 **amplitude** for EM than BA (*t*(17) = 3.49, *p* = 0.008, *d* = 0.79) and LA (*t*(17) = 4.51, *p* < 0.001, *d* = 0.65). No difference reached significance at P7.

N170 **latency** was modulated by the main effect of system (P7, *F*(2, 34) = 26.12, *p* < 0.001, η_p_^2^ = 0.61; P8, *F*(2, 34) = 30.12, *p* < 0.001, η_p_^2^ = 0.64). It was shorter for EM than LA (P7, *t*(17) = 6.29, *p* < 0.001, *d* = 1.85; P8, *t*(17) = 6.06, *p* < 0.001, *d* = 1.83) and BA (P7, *t*(17) = 6.15, *p* < 0.001, *d* = 1.79; P8, *t*(17) = 5.36, *p* < 0.001, *d* = 1.69) without LA-BA difference.

### N200 & P300 results

Like the face perception task, the active auditory oddball task did not give rise to differences between the systems regarding response speed (EM-LA, *V* = 89, *p* = 0.571; EM-BA, *V* = 116, *p* = 0.701; LA-BA, *V* = 100, *p* = 0.869) nor speed variability (EM-LA, *V* = 83, *p* = 0.430; EM-BA, *V* = 105, *p* = 0.999; LA-BA, *V* = 101, *p* = 0.898).

**Figure 5:**
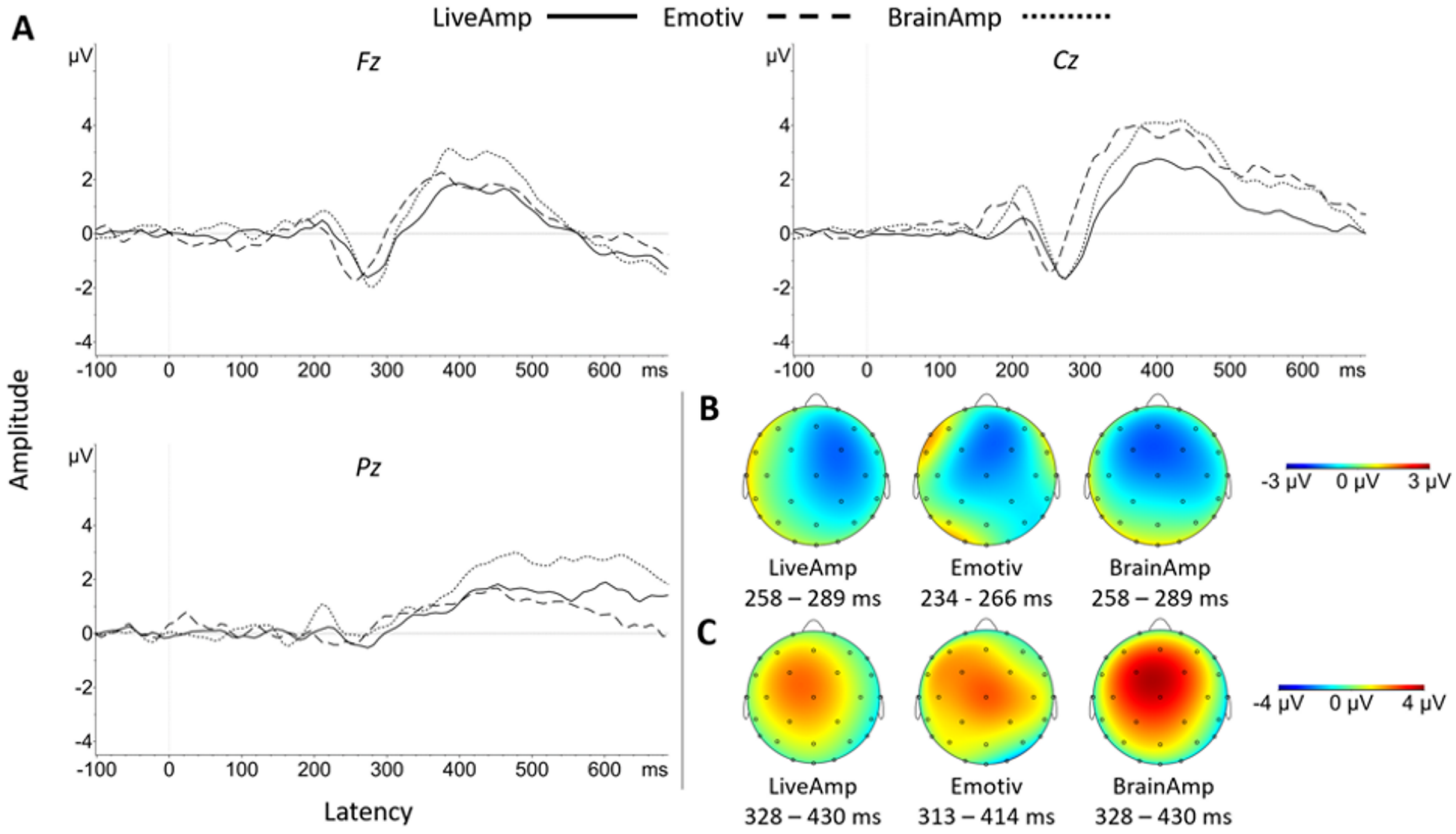
Group N200 and P300 waveforms for the three systems at Fz, Cz and Pz electrodes (A). Topographic distribution of N200 (B) and P300 (C) ERP signals for the three systems.

The **N200 amplitude** in Pack-100 and native sampling rate significantly differed from zero for all three systems (Table 8). It was large in size for BA (medium in size at Pz) and LA, and medium in size for EM, with prominence at Fz for every system (Fig. 5). Despite reduced datasets (Packs-75, 50) for every system or lowered sampling rate (LA and BA), the N200 effect size remained large and prominent at Fz. N200 amplitude was not significantly affected by a main effect of system in Packs-100a, 100b, 75 and 50, at Fz, Cz or Pz (all *p*_s_ > 0.415).

**Table 8:** Peak latency of N200, and comparisons of the N200 amplitude to zero for each pack, sampling rate, system, and electrode site.

Data set		EEG system	Site	Peak latency, ms (SE)	Amplitude, mV (SE)	*t*-value *(df = 17)*	*p-value*	Cohen’s *d*
Pack	Sampling							
100a	128 Hz	EM	Fz	253.91 (4.25)	2.16 (0.71)	3.05	0.007**	0.72
			Cz	253.91 (3.24)	2.00 (0.81)	2.47	0.024*	0.58
			Pz	240.02 (5.20)	1.35 (0.43)	3.13	0.006**	0.74
		LA	Fz	263.89 (6.40)	2.11 (0.50)	4.20	<0.001***	0.99
			Cz	258.25 (6.90)	2.32 (0.63)	3.69	0.002**	0.87
			Pz	248.70 (8.96)	1.46 (0.31)	4.75	<0.001***	1.12
		BA	Fz	278.21 (3.69)	2.75 (0.43)	6.42	<0.001***	1.51
			Cz	271.70 (4.71)	2.54 (0.63)	4.02	<0.001***	0.95
			Pz	259.55 (6.70)	1.34 (0.39)	3.45	0.003**	0.81
100b	500 Hz	LA	Fz	259.89 (6.07)	2.05 (0.46)	4.43	<0.001***	1.05
			Cz	257.78 (6.29)	2.14 (0.59)	3.63	0.002**	0.86
			Pz	245.22 (6.85)	1.42 (0.33)	4.39	<0.001***	1.03
	250 Hz	BA	Fz	270.00 (2.18)	2.32 (0.45)	5.18	<0.001***	1.22
			Cz	258.67 (5.44)	2.26 (0.55)	4.07	<0.001***	0.96
			Pz	246.89 (5.52)	1.18 (0.38)	3.10	0.006**	0.73
75	128 Hz	EM	Fz	248.70 (4.34)	2.33 (0.58)	4.01	<0.001***	0.95
			Cz	251.74 (3.77)	1.87 (0.85)	2.21	0.041*	0.52
			Pz	242.62 (5.70)	1.22 (0.39)	3.11	0.006**	0.73
	500 Hz	LA	Fz	257.56 (6.73)	2.25 (0.51)	4.38	<0.001***	1.03
			Cz	253.67 (7.03)	2.31 (0.64)	3.60	0.002**	0.85
			Pz	246.89 (6.11)	1.60 (0.40)	4.04	<0.001***	0.95
	250 Hz	BA	Fz	269.56 (2.42)	2.68 (0.50)	5.32	<0.001***	1.25
			Cz	257.33 (5.61)	2.62 (0.62)	4.21	<0.001***	0.99
			Pz	248.44 (5.05)	1.20 (0.46)	2.59	0.019*	0.61
50	128 Hz	EM	Fz	252.17 (4.13)	2.47 (0.66)	3.76	0.002**	0.89
			Cz	257.81 (3.74)	2.45 (1.05)	2.33	0.033*	0.55
			Pz	239.59 (5.69)	1.71 (0.57)	3.00	0.008**	0.71
	500 Hz	LA	Fz	256.78 (6.17)	2.19 (0.57)	3.86	0.001**	0.91
			Cz	257.89 (6.94)	2.61 (0.71)	3.70	0.002**	0.87
			Pz	245.78 (7.12)	1.87 (0.42)	4.42	<0.001***	1.04
	250 Hz	BA	Fz	263.33 (4.59)	2.69 (0.55)	4.86	<0.001***	1.14
			Cz	257.56 (5.53)	2.89 (0.63)	4.62	<0.001***	1.09
			Pz	250.89 (5.93)	1.24 (0.50)	2.48	0.024*	0.58

**Notes:**

* *p* ≤ 0.05.

** *p* ≤ 0.01.

*** *p* ≤ 0.001.

Regarding **N200 latency**, there was no significant main effect of system at the native sampling rate (Pack-100b) (Fz, *F*(2, 34) = 3.50, *p* = 0.064, η_p_^2^ = 0.17; Cz, *F*(2, 34) = 0.51, *p* = 0.606; Pz, *F*(2, 34) = 0.90, *p* = 0.416). The marginal effect at Fz site reflected shorter latency for EM than BA (*t*(17) = 4.67, *p* < 0.001, *d* = 1.12). A main effect of system appeared with a lower sampling rate (Pack-100a: Fz, *F*(2, 34) = 7.66, *p* = 0.006, η_p_^2^ = 0.31; Cz, *F*(2, 34) = 5.73, *p* = 0.016, η_p_^2^ = 0.25; Pz, *F*(2, 34) = 3.51, *p* = 0.041, η_p_^2^ = 0.17), reflecting significantly shorter latency with EM than BA (Fz, *t*(17) = 6.51, *p* < 0.001, *d* = 1.44; Cz, *t*(17) = 5.25, *p* < 0.001, *d* = 1.04; Pz, *t*(17) = 3.07, *p* = 0.021, *d* = 0.77). For Pack-75, the system effect was significant at Fz site (*F*(2, 34) = 4.77, *p* = 0.031, η_p_^2^ = 0.22) reflecting the EM < BA pattern (*t*(17) = 5.69, *p* < 0.001, *d* = 1.40). For Pack-50, there was no significant main effect of system, but the EM < BA pattern remained at Fz (*t*(17) = 2.84, *p* = 0.034, *d* = 0.60).

**P300 amplitude** significantly exceeded zero at Fz and Cz, with a large effect size for every pack and system (Table 9). It was prominent at Cz (except with LA in Packs-75 and 50) and overall smaller in size, but still significant, at Pz.

**Table 9:** P300 amplitude and peak latency comparisons to zero, for each pack, sampling rate, system, and electrode site.

Data set		EEG system	Site	Peak latency, ms (SE)	Amplitude, mV (SE)	*t*-value *(df = 17)*	*p*-value	Cohen’s *d*
Pack	Sampling							
100a	128 Hz	EM	Fz	360.24 (7.94)	3.51 (0.90)	3.92	<0.001***	0.92
			Cz	363.72 (9.14)	5.54 (1.10)	5.06	<0.001***	1.19
			Pz	358.94 (12.19)	2.69 (0.86)	3.12	0.006**	0.74
		LA	Fz	385.42 (7.61)	2.85 (0.68)	4.18	<0.001***	0.99
			Cz	383.25 (10.93)	3.80 (0.76)	5.02	<0.001***	1.18
			Pz	358.51 (14.47)	2.36 (0.65)	3.63	0.002**	0.86
		BA	Fz	373.26 (7.51)	4.12 (0.66)	6.28	<0.001***	1.48
			Cz	378.47 (9.87)	5.53 (0.67)	8.25	<0.001**	1.94
			Pz	378.47 (11.16)	3.03 (0.56)	5.38	<0.001**	1.27
100b	500 Hz	LA	Fz	381.89 (7.40)	2.88 (0.61)	4.75	<0.001***	1.12
			Cz	383.11 (9.51)	3.60 (0.76)	4.76	<0.001***	1.12
			Pz	359.33 (12.22)	2.14 (0.59)	3.60	0.002**	0.85
	250 Hz	BA	Fz	381.11 (7.62)	4.21 (0.65)	6.49	<0.001***	1.53
			Cz	391.33 (6.76)	5.53 (0.65)	8.46	<0.001***	1.99
			Pz	384.67 (9.77)	3.14 (0.69)	4.53	<0.001***	1.07
75	128 Hz	EM	Fz	352.43 (7.11)	3.35 (0.93)	3.58	0.002**	0.85
			Cz	360.24 (9.98)	5.75 (1.19)	4.85	<0.001***	1.14
			Pz	361.11 (11.66)	2.69 (0.76)	3.52	0.003**	0.83
	500 Hz	LA	Fz	371.22 (8.31)	2.96 (0.61)	4.89	<0.001***	1.15
			Cz	380.78 (9.84)	3.88 (0.81)	4.78	<0.001***	1.13
			Pz	363.11 (11.86)	2.45 (0.70)	3.50	0.003**	0.83
	250 Hz	BA	Fz	384.44 (7.52)	4.22 (0.65)	6.52	<0.001***	1.54
			Cz	381.33 (7.99)	5.73 (0.76)	7.52	<0.001***	1.77
			Pz	379.78 (11.20)	3.32 (0.77)	4.29	<0.001***	1.01
50	128 Hz	EM	Fz	355.47 (7.58)	3.90 (0.98)	3.99	<0.001***	0.94
			Cz	361.11 (11.27)	5.34 (1.30)	4.11	<0.001***	0.97
			Pz	362.85 (11.70)	2.77 (0.70)	3.97	<0.001***	0.94
	500 Hz	LA	Fz	375.22 (9.16)	3.13 (0.55)	5.68	<0.001***	1.34
			Cz	379.89 (9.74)	3.68 (0.78)	4.70	<0.001***	1.11
			Pz	366.44 (12.49)	2.36 (0.77)	3.08	0.003**	0.73
	250 Hz	BA	Fz	372.67 (8.36)	4.14 (0.64)	6.48	<0.001***	1.53
			Cz	386.89 (6.98)	5.39 (0.76)	7.13	<0.001***	1.68
			Pz	390.89 (8.66)	3.45 (0.81)	3.64	0.001**	0.86

**Notes:**

** *p* ≤ 0.01.

*** *p* ≤ 0.001.

These was no main effect of system on P300 amplitude in all Packs and electrodes.

In Pack-100b, the main effect of system was significant on **P300 latency** (Fz, *F*(2, 34) = 3.90, *p* = 0.030, η_p_^2^ = 0.19; Cz, *F*(2, 34) = 4.57, *p* = 0.018, η_p_^2^ = 0.21), reflecting shorter latency for EM than BA (Fz, *t*(17) = 2.75, *p* = 0.041, *d* = 0.63; Cz, *t*(17) = 3.18, *p* = 0.016, *d* = 0.81) and LA at Fz (*t*(17) = 2.71, *p* = 0.045, *d* = 0.66). In Pack-100a, the main effect of system at Fz (*F*(2, 34) = 5.57, *p* = 0.008, η_p_^2^ = 0.25) was explained by a shorter latency for EM than LA (*t*(17) = 2.82, *p* = 0.035, *d* = 0.76). The system effect at Fz was replicated in Pack-75 (*F*(2, 34) = 7.87, *p* = 0.002, η_p_^2^ = 0.32), with an EM < BA pattern (*t*(17) = 5.29, *p* < 0.001, *d* = 1.03), but there was no system effect in Pack-50.

### MMN results

**Figure 6:**
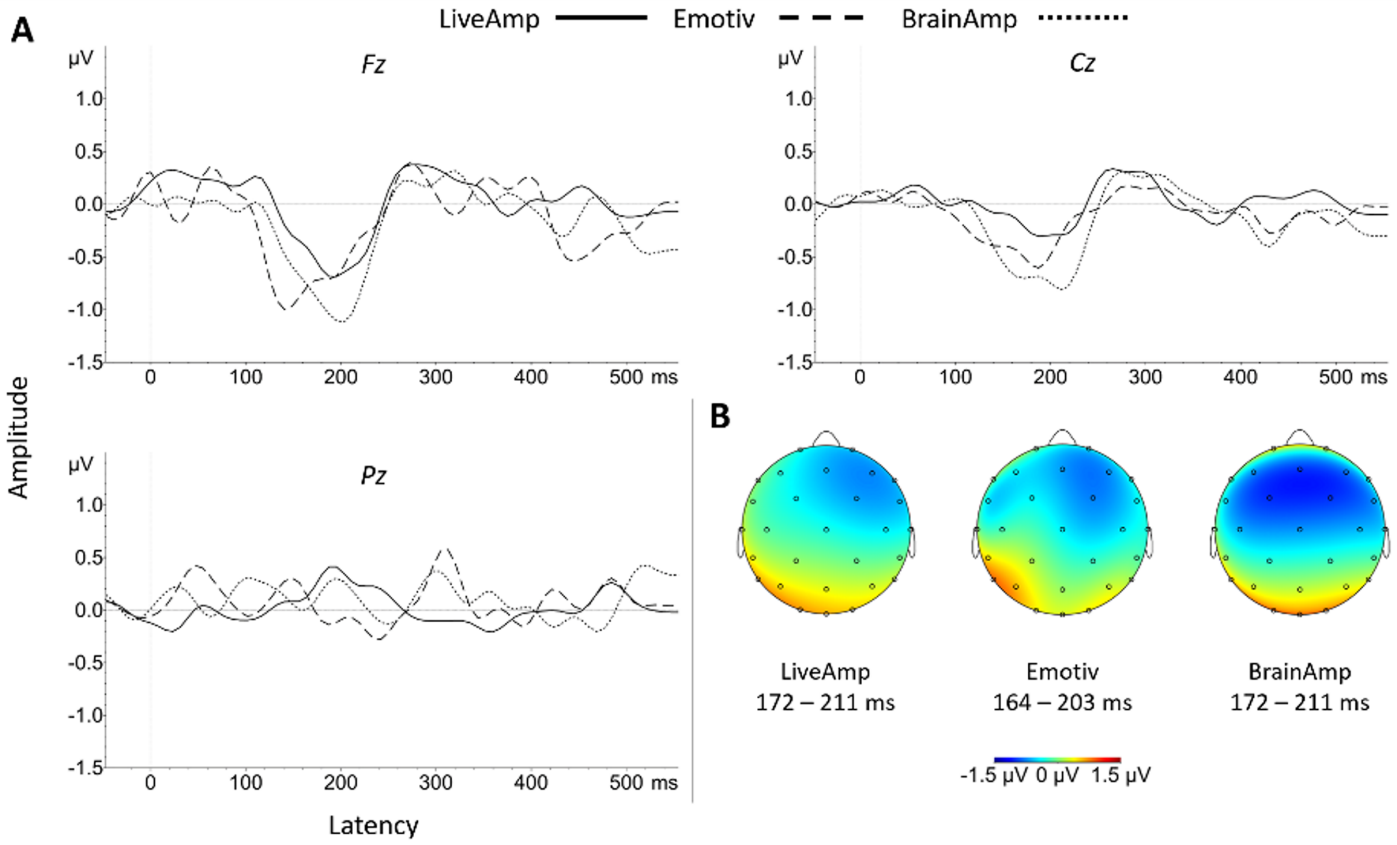
(A) Group MMN ERP waveforms for the three systems at Fz, Cz and Pz electrode sites. (B) Topographic distributions of MMN ERP signals for each system.

The AUC of the MMN in Pack-100 at native sampling rate significantly exceeded zero for every system at Fz (Table 10); the effect size was large (LA, BA), or medium (EM). It also exceeded zero significantly at Cz, medium-sized for LA and BA and small for EM (Fig. 6), while it was not significant at Pz. It remained the highest at Fz, even in Pack-100a, for all three systems.

**Table 10:** Peak latency of the MMN, Area Under the Curve of MMN (AUC), and comparisons between MMN amplitude to zero (*t*-tests) at Fz, Cz and Pz electrodes, for the three EEG systems, each pack and sampling rate.

Data set		EEG System	Electrode	MMN peak latency, ms (*SE*)	AUC of MMN, nV.s (*SE*)	*t*-value (*df* = 17)	*p*-value	Cohen’s *d*
Pack	Sampling							
100a	128 Hz	EM	Fz	179.26 (6.84)	−42.31 (13.10)	3.23	0.002**	0.76
			Cz	193.58 (8.94)	−22.87 (11.92)	1.92	0.036*	0.45
			Pz	209.20 (7.02)	7.39 (12.12)	0.61	0.725	0.14
		LA	Fz	193.58 (5.74)	−32.58 (7.04)	4.63	<0.001***	1.09
			Cz	190.54 (6.63)	−15.52 (5.93)	2.62	0.009**	0.62
			Pz	195.31 (9.22)	16.50 (5.78)	2.85	0.994	0.67
		BA	Fz	186.63 (5.75)	−50.09 (10.23)	4.90	<0.001***	1.15
			Cz	189.24 (6.96)	−37.21 (11.05)	3.37	0.002**	0.79
			Pz	188.81 (8.48)	8.43 (7.36)	1.15	0.866	0.27
100b	500 Hz	LA	Fz	193.22 (5.97)	−30.25 (7.83)	3.86	<0.001***	0.91
			Cz	195.78 (6.74)	−14.08 (5.95)	2.37	0.015*	0.56
			Pz	202.56 (9.50)	15.91 (6.22)	2.56	0.990	0.60
	250 Hz	BA	Fz	191.78 (5.82)	−48.63 (10.19)	4.77	<0.001***	1.13
			Cz	190.89 (6.51)	−34.63 (10.88)	3.18	0.003**	0.75
			Pz	207.78 (9.16)	11.05 (7.69)	1.44	0.916	0.34
75	128 Hz	EM	Fz	178.82 (6.56)	−47.37 (11.48)	4.13	<0.001***	0.97
			Cz	197.05 (8.44)	−24.57 (11.50)	2.14	0.024*	0.50
			Pz	207.47 (7.13)	4.26 (12.34)	0.35	0.633	0.08
	500 Hz	LA	Fz	185.33 (5.64)	−28.91 (7.87)	3.67	<0.001***	0.87
			Cz	191.56 (6.38)	−19.46 (5.98)	3.25	0.002**	0.77
			Pz	203.00 (9.25)	9.64 (5.59)	1.72	0.949	0.41
	250 Hz	BA	Fz	194.89 (5.77)	−50.49 (9.97)	5.07	<0.001***	1.19
			Cz	193.33 (6.38)	−36.19 (12.44)	2.91	0.005**	0.69
			Pz	206.22 (8.89)	9.51 (8.79)	1.08	0.853	0.25
50	128 Hz	EM	Fz	185.77 (7.65)	−56.22 (14.10)	3.99	<0.001***	0.94
			Cz	194.45 (8.13)	−28.37 (13.98)	2.03	0.029*	0.48
			Pz	205.30 (7.06)	5. 5.84 (16.65)	0.35	0.635	0.08
	500 Hz	LA	Fz	186.11 (6.13)	−30.34 (9.88)	3.07	0.003**	0.72
			Cz	193.44 (6.39)	−16.38 (7.22)	2.27	0.018*	0.53
			Pz	201.67 (8.59)	9.94 (6.68)	1.49	0.923	0.35
	250 Hz	BA	Fz	192.44 (5.24)	−51.72 (10.86)	4.76	<0.001***	1.12
			Cz	190.22 (6.13)	−37.67 (13.81)	2.73	0.007**	0.64
			Pz	197.11 (8.41)	14.45 (11.15)	1.30	0.894	0.31

**Notes:**

* *p* ≤ 0.05.

** *p* ≤ 0.01.

*** *p* ≤ 0.001.

For BA, over Packs-100b, 100a, 75 and 50, the significant MMN **amplitude** was medium in size at Cz, and large at Fz. For LA, the significant MMN was medium in size over every pack at Cz, whereas it decreased sharply from large over Pack-100b, 100a and 75 towards medium size in Pack-50 at Fz. For EM, at Cz, the significant MMN was small in Pack-100a and -50, but medium in Pack-75, while it increased from medium size in Pack-100a to large size in Pack-75 and -50 at Fz. No main effect of system, and no two-by-two system comparison reached significance, whatever the sampling rate or the pack, at any electrode site.

There was no system effect on the MMN **latency**, peaking around 180–200 ms for every dataset (Table 10). Detailed two-by-two comparisons did not reveal any significant difference.

### Between-system correlations

Correlations calculated to assess measurement consistency across participants between the three systems (LA/BA, EM/LA, and EM/BA) are detailed in Table 11. Theses correlations reached significance (*p* < 0.05) when exceeding *r* = 0.47. With respect to time/frequency measures (alpha-power and SSVEP), most correlations were significant or near-significant (even with reduced data-sets), with the notable exception of the EM-BA relation for 15 Hz condition. With respect to ERP components, the systems were highly correlated for both the N170 amplitude and latency at P7, but only for latency at P8. For the N200 as P300, few correlations were significant for latencies, but correlations were largely significant for amplitude. Taken together, various indices supported relatively high measurement consistency between participants for time/frequency measures across the systems, and moderate consistency for ERP components with the exception of the MMN, (see Figs. S1 to S6).

**Table 11:** Across participants correlation values (Pearson) between the measures (time/frequency or ERPs) recorded with the three systems (EM, LA, BA) in every task and in every data-set (Pack).

Task	Condition	Electrode	Comparison	Pack			
				100a	100b	75	50
Resting state (alpha)			LA-BA	–	0.80	0.82	0.84
			EM-LA	–	0.50	0.49	0.51
			EM-BA	–	0.43	0.47	0.41
SSVEP	6 Hz		LA-BA	–	0.88	0.79	0.69
			EM-LA	–	0.90	0.81	0.70
			EM-BA	–	0.88	0.91	0.87
	10 Hz		LA-BA	–	0.80	0.74	0.67
			EM-LA	–	0.44	0.46	0.46
			EM-BA	–	0.50	0.55	0.41
	15 Hz		LA-BA	–	0.75	0.69	0.69
			EM-LA	–	0.60	0.53	0.54
			EM-BA	–	0.30	0.32	0.30
Face perception N170	Amplitude	P7	LA-BA	0.77	0.78	0.77	0.79
			EM-LA	0.75	0.73	0.71	0.71
			EM-BA	0.73	0.74	0.72	0.74
		P8	LA-BA	0.58	0.59	0.62	0.61
			EM-LA	0.37	0.37	0.35	0.32
			EM-BA	0.36	0.39	0.40	0.42
	Latency	P7	LA-BA	0.80	0.80	0.82	0.77
			EM-LA	0.81	0.72	0.66	0.44
			EM-BA	0.53	0.70	0.64	0.66
		P8	LA-BA	0.88	0.95	0.98	0.77
			EM-LA	0.83	0.77	0.71	0.67
			EM-BA	0.74	0.78	0.65	0.54
Active auditory oddball N200	Amplitude	Fz	LA-BA	0.59	0.55	0.67	0.69
			EM-LA	0.62	0.71	0.54	0.43
			EM-BA	0.53	0.58	0.47	0.31
		Cz	LA-BA	0.89	0.81	0.81	0.74
			EM-LA	0.62	0.60	0.57	0.51
			EM-BA	0.72	0.76	0.71	0.54
	Latency	Fz	LA-BA	0.14	0.08	0.15	0.10
			EM-LA	0.07	0.06	0.07	0.09
			EM-BA	0.57	0.59	0.54	0.60
		Cz	LA-BA	0.42	0.70	0.35	0.03
			EM-LA	0.50	0.56	0.07	0.03
			EM-BA	0.69	0.37	0.33	0.12
Active auditory oddball P300	Amplitude	Fz	LA-BA	0.62	0.49	0.64	0.59
			EM-LA	0.63	0.56	0.61	0.28
			EM-BA	0.45	0.45	0.43	0.45
		Cz	LA-BA	0.72	0.76	0.77	0.74
			EM-LA	0.49	0.47	0.52	0.39
			EM-BA	0.57	0.56	0.60	0.60
	Latency	Fz	LA-BA	0.49	0.02	0.25	0.31
			EM-LA	0.34	0.46	0.46	0.28
			EM-BA	0.73	0.53	0.66	0.59
		Cz	LA-BA	0.49	0.24	−0.20	−0.01
			EM-LA	0.52	0.53	0.48	−37
			EM-BA	0.39	0.44	0.38	−58
Passive auditory oddball MMN	Amplitude	Fz	LA-BA	0.30	0.48	0.20	0.28
			EM-LA	0.26	0.18	0.17	0.32
			EM-BA	0.07	−0.05	−0.17	0.24
		Cz	LA-BA	0.33	0.35	0.14	0.23
			EM-LA	0.36	0.41	0.45	0.06
			EM-BA	0.54	0.42	0.33	0.30
	Latency	Fz	LA-BA	0.11	0.02	0.03	−0.18
			EM-LA	0.27	0.19	0.43	0.27
			EM-BA	0.21	0.23	−0.09	−0.26
		Cz	LA-BA	0.00	0.06	−0.14	0.21
			EM-LA	−0.07	−0.14	0.54	0.08
			EM-BA	−0.03	−0.05	−0.22	−0.13

### Standardized measurement error

Data are shown in Table 12. Key findings were (1) systematically lower SME for the LA system measuring MMN, N170, N2 and P3 amplitude (LA < BA < EM), (2) no difference between systems when measuring latencies except the EM > LA = BA pattern at P8 and the EM > BA at P7 for the N170, (3) regarding the N170, each system had a lower SME than the ERP CORE dataset for both amplitude and latency, whereas EM exhibited a higher SME that the ERP CORE, (4) as to the MMN latency, higher SME was observed for each of the three systems as compared with the ERP CORE dataset (see Tables S1, S2 and Fig. S7).

**Table 12:** Between-system comparisons of the standardized measurement error (SME) for each ERP component for Pack-100b. *Post-hoc* comparisons are not reported when Friedman test is not significant.

ERP	Measure	Electrode	Friedman: df1, df2, *p*	Comparison	Wilcoxon
N170	Amplitude	P7	*Q*(1.9, 34.1) = 12.36, *p* < 0.001***	BA-EM	*W* = 80, *p* > 0.999
				BA-LA	*W* = 10, *p* < 0.001***
				LA-EM	*W* = 20, *p* = 0.004**
		P8	*Q*(1.9, 34.1) = 9.08, *p* < 0.001***	BA-EM	*W* = 71, *p* > 0.999
				BA-LA	*W* = 1, *p* < 0.001***
				LA-EM	*W* = 63, *p* = 0.630
	Latency	P7	*Q*(1.9, 34.1) = 9.17, *p* < 0.001***	BA-EM	*W* = 18, *p* = 0.003**
				BA-LA	*W* = 60, *p* = 0.830
				LA-EM	*W* = 39, *p* = 0.070
		P8	*Q*(1.9, 34.1) = 24.04, *p* < 0.001***	BA-EM	*W* = 3, *p* < 0.001***
				BA-LA	*W* = 73, *p* > 0.999
				LA-EM	*W* = 1, *p* < 0.001***
N200	Amplitude	Fz	*Q*(1.9, 32.1) = 19.24, *p* < 0.001***	BA-EM	*W* = 3, *p* < 0.001***
				BA-LA	*W* = 37, *p* = 0.103
				LA-EM	*W* = 3, *p* < 0.001***
		Cz	*Q*(1.9, 32.1) = 49.36, *p* < 0.001***	BA-EM	*W* = 11, *p* = 0.001***
				BA-LA	*W* = 12, *p* = 0.002**
				LA-EM	*W* = 0, *p* < 0.001***
	Latency	Fz	*Q*(1.9, 32.1) = 2.13, *p* = 0.138		
		Cz	*Q*(1.9, 32.1) = 2.59, *p* = 0.093		
P300	Amplitude	Fz	*Q*(1.9, 32.1) = 19.24, *p* < 0.001***	BA-EM	*W* = 9, *p* < 0.001***
				BA-LA	*W* = 33, *p* = 0.062
				LA-EM	*W* = 1, *p* < 0.001***
		Cz	*Q*(1.9, 32.1) = 49.36, *p* < 0.001***	BA-EM	*W* = 10, *p* < 0.001***
				BA-LA	*W* = 10, *p* < 0.001***
				LA-EM	*W* = 0, *p* < 0.001***
	Latency	Fz	*Q*(1.9, 32.1) = 4.10, *p* = 0.028*	BA-EM	*W* = 46, *p* = 0.269
				BA-LA	*W* = 69, *p* > 0.999
				LA-EM	*W* = 74, *p > 0*.999
		Cz	*Q*(1.9, 32.1) = 1.06, *p* = 0.355		
MMN	AUC	Fz	*Q*(1.9, 32.1) = 31.74, *p* < 0.001***	BA-EM	*W* = 12, *p* = 0.002**
				BA-LA	*W* = 17, *p* = 0.009**
				LA-EM	*W* = 5, *p* < 0.001***
		Cz	*Q*(1.9, 32.1) = 105.40, *p* < 0.001***	BA-EM	*W* = 13, *p* = 0.002**
				BA-LA	*W* = 0, *p* < 0.001***
				LA-EM	*W* = 0, *p* < 0.001***
	Latency	Fz	*Q*(1.9, 32.1) = 0.71, *p* = 0.491		
		Cz	*Q*(1.9, 32.1) = 0.38, *p* = 0.678		

**Notes:**

* *p* ≤ 0.05.

** *p* ≤ 0.01.

*** *p* ≤ 0.001.

### Effect sizes for every system, according to task duration/number of trials

Table 13 provides an overview of the effect sizes for each task, system and Pack (expressed in seconds or number of trials). This synthesized presentation meets our objective regarding guidelines for researchers.

**Table 13:** Effect size (Cohen’s *d*) of the expected spectral features of the neural signal as compared to zero in two paradigms (Resting State, SSVEP) and of the four studied ERPs, as a function of the size of the dataset (Pack-100%, Pack75%, Pack 50%) and the EEG system. For SSVEP, *d* values for the fundamental frequency and its 2 first harmonics have been averaged; for N170, *d* values in face condition have been averaged over P7 and P8; for N200, P300 and MMN, *d* values have been averaged over Fz, Cz, and Pz. Duration or number of trials are presented on the gray lines (s = second).

	Resting state	SSVEP	N170 (Peak-to-Peak)	N200 (peak amplitude)	P300 (peak amplitude)	MMN (AUC)
Pack-100	60 s	30 s	300 trials (150 faces)	400 trials (80 deviants)		674 trials (112 deviants)
EM	[1.03]	[1.45]	[1.23]	[0.68]	[0.95]	[0.45]
LA	[1.79]	[2.12]	[1.54]	[0.98]	[1.03]	[0.69]
BA	[1.24]	[1.67]	[1.86]	[0.97]	[1.53]	[0.74]
Pack-75	45 s	22,5 s	225 trials (112 faces)	300 trials (60 deviants)		505 trials (84 deviants)
EM	[1.06]	[1.39]	[1.22]	[0.73]	[0.94]	[0.52]
LA	[1.80]	[1.61]	[1.56]	[0.94]	[1.03]	[0.68]
BA	[1.18]	[2.09]	[1.81]	[0.95]	[1.44]	[0.71]
Pack-50	30 s	15 s	150 trials (75 faces)	200 trials (40 deviants)		337 trials (56 deviants)
EM	[1.15]	[1.46]	[1.16]	[0.71]	[0.95]	[0.50]
LA	[1.76]	[1.58]	[1.60]	[0.94]	[1.06]	[0.54]
BA	[1.20]	[2.25]	[1.79]	[0.94]	[1.36]	[0.69]

## Discussion

We aimed to compare how three EEG systems—a mobile consumer-grade system (EM), a portable research-grade system (LA), and a non-portable research-grade system (BA)—perform on measures of well-established electrophysiological indices of mental states, sensory, and cognitive processes. All three systems succeeded in showing, (1) alpha power changes in resting state, (2) spectral variations guided by the frequency of flickering stimuli, (3) ERP responses to faces (N170), and to controlled (N200, P300) or automatic (MMN) auditory ERPs. Therefore, the results support our three hypotheses and highlight how effective but also limited may be wearable EEG systems.

### Spectral analyses

Consistent with our hypothesis, all three EEG systems captured the occipital Berger Effect already observed in adults (Johnstone, Blackman & Bruggemann, 2012; Cannard, Wahbeh & Delorme, 2021; An et al., 2022) and children (Barry et al., 2009; Bhavnani et al., 2022; Edgar et al., 2023). The effect was stable and large in size even in the 30-s Pack-50 with every system, though it was of larger size with LA and BA compared to the consumer-grade EM system. These differences in spectral responses could not be attributed to discrepancy between recording resolution, as the three systems were equated in sampling rate. Though the Berger Effect has previously been reported with very minimalist devices (Johnstone, Blackman & Bruggemann, 2012; Debener et al., 2015; Shivaraja et al., 2023), mixed results were obtained from a 14-electrode EM system (Grummett et al., 2015; Mahdid, Lee & Blain-Moraes, 2020; Kounios et al., 2024). In the current study, the 32-electrode mobile EM system proved effective in assessing oscillations and succeeded in demonstrating the Berger Effect.

We also found that flickering stimuli evoked oscillatory brain responses exactly at the periodic stimulation frequency and its two first harmonics (SSVEP) across every EEG system. It should be noted that, with LA, these effects were large in size even on a short recording (15-s), highlighting the efficiency of this portable research-grade system in assessing oscillations, but a drop in effect size was however observed in Packs-75 and -50 compared to Pack-100 for LA, not for BA. Consequently, using only 15- or 22.5-s recording, comparable effect sizes were obtained with LA and EM systems, in line with similar spectral effects shown using EM and a medical-grade EEG system during emotional clips perception (Katsigiannis & Ramzan, 2018).

Despite showing lower SNR responses than LA and BA, the EM system revealed effective for spectral analyses, probably due to the choice of wet electrodes for EM and LA. Indeed, systems with dry electrodes have been found less efficient (Xing et al., 2018; Ladouce et al., 2022). However, there are two restrictions. Firstly, for SSVEP, increasing the dataset duration up to 30 s led to higher effect size with LA, but not EM. Therefore, longer stimulation did not compensate for less exploitable signal from EM. Secondly, EM captured the expected SNR increase at the 6 or 15 Hz fundamental frequency of a periodic visual stimulation and its harmonics, but it failed to do so in response to 10 Hz-stimulation, contrary to LA and BA. This may come from a temporal instability of SSVEP responses, as a decline of amplitude specifically after 10 and 15 Hz has been documented (Łabęcki et al., 2024). Another possibility is that 10 Hz is part of the alpha range (8–12 Hz), which is vulnerable to changes in endogenous attentional states. Endogenous attention has been shown to interfere with SNR variations and decrease their amplitude in response to flickers at alpha rhythm (*e.g*., 10 Hz) (Gulbinaite, Roozendaal & VanRullen, 2019). To avoid alpha wave contamination, slightly higher rates (12–13 Hz) are preferred in SSVEP experiments (Yang, Paller & Van Vugt, 2022). In sum, EM was found to record satisfactory SSVEP signature, but caution is advised when EM is used to assess the brain’s ability to discriminate images below the threshold of consciousness.

### ERP analyses

Compared to spectral aspects, ERPs require time/amplitude analyses on very short signal durations, and the ability of consumer-grade systems to do so has been rarely questioned. Below, we discuss our results with respect to the N170, N200, P300, and MMN.

First, all three EEG systems showed a large N170, peaking at its typical posterior site (P7 and P8) in the face condition, with a much smaller amplitude, texture conditions, consistent with its well-known association with face perception (Bentin et al., 1996). This N170 was robust enough to remain large after LA and BA downsampling, or dataset reduction (*e.g*., Pack-50, 150 trials). The EM system detected an N170 that was only slightly smaller than with BA (but comparable to the LA system, except in the very short 150-trials dataset). Its efficiency in detecting the N170 was in line with previous studies examining similar systems (De Lissa et al., 2015; Soto et al., 2018; Wehrman et al., 2021; Sagehorn et al., 2024). Taken together, the results contribute to validate the use of mobile, wireless and low-cost EEG systems for research about N170. Note, however, that the posterior N170 prominence was less typical from EM than from LA and BA. Additionally, an overall reduction in latency occurred using EM for most of the ERP components, including N170. EM users are therefore encouraged to focus on ERP amplitude, and should be aware of shortcomings regarding spatial distribution and temporal precision.

Second, although the N200 has not always been obtained in auditory oddball studies (Falkenstein, Hoormann & Hohnsbein, 1999, 2002; Smith, Johnstone & Barry, 2004), all three EEG systems were able to detect it as a medium-to-large cerebral response with consistent frontal prominence in our auditory oddball task, despite dataset reduction for every system and sampling rate lowering (LA and BA). N200 amplitude did not significantly differ between systems. This effectiveness of the EM system was consistent with a previous study (Barham et al., 2017). Like the N170, the N200 peaked earlier using the EM than the BA system, reflecting lower time precision for EM.

Third, the P300 also appeared as large-sized with the three systems at the expected fronto-central site, replicating (Badcock et al., 2015). LA contrasted with EM and BA by failing to show Cz prominence for P300 in short datasets (Packs-75 and -50). EM and BA systems succeeded in recording P300 of similar size and were unaffected by dataset reductions. The efficiency of the EM system here contrasts with a previous study comparing EM with a standard system (Duvinage et al., 2013). However, it is worth noting that this previous study, which evaluated EM for medical diagnosis, differed in several aspects from ours (*e.g*., participants were walking, deviants were frequent, and EM was compared to a medical-grade EEG device). Nevertheless, like the N200 and N170, the P300 appeared earlier with EM than LA and BA.

Fourth, in line with our hypothesis, a significant fronto-central MMN was observed across all systems and all sampling rates, with stable prominence in effect size at Fz over Cz and Pz. At Fz site, the MMN was stable in amplitude (medium to large) over all systems, in line with MMN comparison with another research-grade system (Neuroscan) (Williams et al., 2020). Mixed results were previously obtained using EM to assess the MMN (Badcock et al., 2013, 2015). Note the decrease in the MMN size with the dataset reduction from Pack-75 (505 trials) to Pack-50 (337 trials) using EM and LA, contrary to BA. As only LA also exhibited sensitivity to the number of trials for P300, users should be aware of the risk of employing few trials to assess ERPs with this system. In contrast with the other investigated components, the MMN was not observed earlier using EM than the other systems. Therefore, the MMN was of satisfactory amplitude, latency, and topography even when recorded with a wireless and consumer grade EEG system.

Overall, one key finding is that the EM system recorded ERPs of reasonable quality. Each targeted ERP component had satisfactory amplitude and intra-individual variability measured by SME reached an appropriate level as compared with standards (Zhang & Luck, 2023). This is in relative contrast with previous reports (Grummett et al., 2015). We speculate that this might be due to the use of a custom acquisition software to improve synchronization between stimuli presentation and EEG recording (Tahmasebzadeh, Bahrani & Setarehdan, 2013). Note, however, that shorter latencies were observed for three of the four assessed ERPs using EM compared to both LA and BA. The observed lack of time precision replicates previous results obtained when the EPOC-Flex was compared to the Biosemi System (Ries et al., 2014). More generally, discrepancies related to latencies have been shown with portable EEG systems (*e.g*., Nautilus) compared with literature data (Melnik et al., 2017), though reports are inconsistent (David Hairston et al., 2014; De Lissa et al., 2015; Williams et al., 2020). Here, we can rule out that this limitation is due to a difference in electrolyte (Ries et al., 2014), because saline solution was used with EM as well as with LA. As a tentative explanation, it can be suggested that time imprecision could be partly due to either the method used to synchronize the EEG data with the events of interest or the internal filters of the EM amplifier. Furthermore, we could find no indication in our results to attribute this difference to a participant effect or task order. In addition, we used robust paradigms adapted from ERP CORE (Kappenman et al., 2021).

In line with Williams, McArthur & Badcock (2021), the low-cost mobile EM system can be recommended for spectrum analyses of EEG signal recorded over relatively long intervals, but it is less precise for time and has to be used with caution to assess some ERPs.

Interestingly, as a portable research-grade system, LA provided robust results with respect to ERP detection. Only minor limitations have been observed regarding LA, (1) for N170, it was less effective than BA in the smallest dataset, and it did not outperform the consumer-grade EM system in terms of effect sizes in larger datasets, (2) slight topographical variability was noticed for P300 in small datasets, (3) the MMN amplitude dropped with the LA and EM systems in the smallest dataset. Some of these weaknesses could be attributed to impedance maintained below 20 kΩ for BA, but only 50 kΩ for LA. Interestingly, no latency difference was observed between ERPs recorded with LA and BA, which suggests a higher temporal precision than with EM.

Taken together, the LA system appears to record reliable signal even with lowered sampling rate, saving energy and memory space, and to be effective except with very few trials.

### General considerations regarding the mobile consumer-grade EM and the portable research-grade LA

Over the last few years, EM has been the prevalent choice for cognitive scientists using consumer-based EEG systems (Sawangjai et al., 2020; Sabio et al., 2024; Gramann, 2024), probably because of its large number of electrodes ensuring wider scalp coverage, and its attractive price. In our study, its signal quality yielded less exploitable data than research-grade systems. Additionally, a major incident (lost amplifier/acquisition computer connection leading to change the headset) occurred twice only with EM. However, with 90% of participants in the final dataset and enough epochs to conduct analyses, EM exhibited overall satisfactory signal quality and can be seen as a reliable tool for researchers, as emphasized in reviews (Lau-Zhu, Lau & McLoughlin, 2019; Niso et al., 2023; Sabio et al., 2024; Jacobsen et al., 2024; Gramann, 2024) and validation studies (Maskeliunas et al., 2016; Melnik et al., 2017; Sawangjai et al., 2020; Khng & Mane, 2020; Williams et al., 2020; Browarska et al., 2021; Williams, McArthur & Badcock, 2021).

In a previous study, Niso et al. (2023) investigated every characteristic of 48 portable systems, highlighting notable advantages of LA over EM system, justifying a price ten times higher for LA than EM. This is consistent with the score for system specifications (CoME S) proposed by Bateson et al. (2017), which considers electrode type, bit resolution, sampling rate, and battery life, on a 4 to 20 scale. According to this scale, LA obtained a higher score with 15-10 than 8-7 for EM. Furthermore, SME measurements have provided evidence for fewer variability in LA data than in the other systems data (often including the BA system). Some higher performance observed from LA over EM system can be attributed to transmission mode (Wi-Fi used for LA is better than Bluetooth used for EM), discrepancies in hardware filter responses, and in amplifiers. Regarding the latter, the bandwidth (Hz) was higher for LA than for EM (131 Hz *vs* 0.16–43 Hz), and the common mode rejection ratio (CMRR) is 80 dB for LA, but it is unknown for EM. Both characteristics could impact the signal amplitude and SNR, which was lower in EM than in LA. The impact of these features, emphasized by Gramann (2024), increases in brief experiments.

More generally, although EM can certainly be used as a research tool (Maskeliunas et al., 2016), it is important to keep in mind that, as well as other consumer-grade systems, EM does not provide raw data and the signal is already preprocessed in untraceable way (Browarska et al., 2021). Despite the exhaustive study by Niso et al. (2023), some technical information is not known and could explain temporal imprecision and slightly lower amplitude of the ERP.

In summary, the portable LA system meets the requirements of a research-grade system with good signal quality and temporal accuracy, but it still underperforms its wired counterpart (BA). Furthermore, although the EM system is definitely more affordable and offers good scalp coverage, as well as a portability score equivalent to that of the LA (CoME D = 3, Niso et al., 2023), it only provides preprocessed data, has a lower SNR, and has shown technical problems such as amplifier-computer disconnections, which need to be taken into account, particularly for time-sensitive measures such as ERPs.

### Limitations

This study contributes to the growing body of research assessing the validity of portable EEG systems to measure neural oscillations and ERPs, and is notable for its analysis of the minimum test duration required to obtain usable data. However, the current findings should be interpreted in light of some limitations.

First, while the sample size was rather large in the context of studies comparing the validity of wireless *vs* wired EEG systems, it remained limited. It might have prevented the detection of small differences between the systems due to insufficient power. Nevertheless, Melnik et al. (2017) calculated that 8% and 9% of variance in ERP measures can be respectively explained by variability between subjects and EEG systems if 16 participants were tested. Thus, our 19-participant group accounted for even smaller part of ERP variance in the present study. It is possible that inter-individual variability may come from contextual differences, since participants were tested at different times of the day, and were not forbidden from drinking coffee or smoking before EEG recordings. For instance, Chen et al. (2020) showed that caffeine intake may enhance the efficiency of individual automatic responses and early cognitive processes. Cui et al. (2020) also showed that abstinence in young smokers may have an impact on the effect of neurophysiological indicators of performance control. However, as shown by the relatively high between participants correlation values observed across systems for time/frequency measures and most ERPs, measures were relatively stable between systems. The lack of correlation for the MMN can tentatively be explained by the specificity of the electrode reference (the common average) used to calculate this ERP component, because of the differential changes in frontal and sub-temporal components of mismatch negativity (Baldeweg, Williams & Gruzelier, 1999).

Second, another limitation concerns comparisons of the SME data for the three systems to the ERP CORE dataset due to few compatibilities between electrode sites of interest. This calculation conveys mitigated conclusions since variability was overall lower than the ERP CORE dataset for the N170 amplitude and latency, while variability was higher than this reference for the MMN latency.

Third, due to a technical problem, the BA system was not used with its optimal sampling rate, which was paradoxically lower than for the LA system. This problem was indirectly solved by analyses conducted with an equalized 128 Hz-sampling rate, initially designed to rule out an interpretation of between-system differences based solely on recording resolution.

Fourth, although researchers may be inclined to use mobile systems, particularly for real-life studies, our study focused on immobile participants. Future research could explore the suitability of these EEG systems in real-world settings, which would involve addressing artifacts caused by large gestures and making informed decisions about preprocessing (Jacobsen et al., 2024). Guidelines on the acceptability of these systems (Bhavnani et al., 2022) and the selection of hardware are available elsewhere (Sawangjai et al., 2020; Niso et al., 2023; Gramann, 2024).

Finally, future studies might extend the comparison of these systems towards tasks involving later cognitive processing, such as sensitivity to semantic or syntactic violations through N400 and P600 components, or addressing spectral power in other bands.

### Practical guidelines

Based on the present study, the following points can be offered as guidelines to researchers wishing to use a portable EEG system to address scientific issues. First, not only the LA, but also the EM consumer-grade EEG system can be recommended if time/frequency analyses on brain oscillations are planned, and no increase in the recording duration is needed. For instance, large changes in the alpha-band, and typical responses to the frequency of flickering stimuli and its harmonics can be observed from 30- and 15-s recordings. Second, whether the system was wired or portable, ERP amplitude did not significantly decrease with the reduction of the number of trials (150, 200 and 300 trials for the N170, N200 and P300, respectively). Except for N200 (and N170 in the texture condition), which can be expected to be of medium size from EM, large-sized ERPs can be recorded using wireless as wired systems. Third, studying ERP latencies is possible with LA, but is compromised by temporal imprecision of EM. Fourth, capturing the MMN with the EM or LA portable systems proved possible, but to a lesser extent than other ERPs, and the number of trials should not be lower than 505 to expect a medium-sized MMN using EM system, while it can be reduced towards 337 using LA or BA.

## Conclusions

The present results were obtained from one fixed and two portable EEG devices outside a specialized EEG recording booth. By replicating several classic ERP components reflecting automatic and voluntary processing of auditory and visual stimuli, as well as neural changes in cerebral rhythms during resting states with open *vs* closed eyes in adults, they provide novel evidence in favor of the validity of such cost-effective, user- and participant-friendly tools. Despite the limitations outlined above, portable EEG systems such as LA and EM may contribute to research in real life conditions. Future comparison studies should be extended to pediatric populations as wireless EEG systems may be particularly useful in developmental research in real-world settings.

## Supplemental Information

10.7717/peerj.20416/supp-1 Supplemental Information 1 Mean bootstrapped standard measurement error (bSME) over all participants for latency and amplitude measures for each system, waveform and electrode site in pack-100b.

10.7717/peerj.20416/supp-2 Supplemental Information 2 Statistical comparisons of the SME values to those obtained with ERP CORE data. * *p* ≤ 0.05, ** *p* ≤ 0.01, *** *p* ≤ 0.001.

10.7717/peerj.20416/supp-3 Supplemental Information 3 Between-system correlations of the mean SNR response to the SSVEP paradigm for each pack and each stimulation condition.

10.7717/peerj.20416/supp-4 Supplemental Information 4 Between-system correlations of the difference in alpha power during the resting state task for each pack.

10.7717/peerj.20416/supp-5 Supplemental Information 5 Between-system correlations of the peak amplitude and latency of the N170 ERP at P7 and P8 for each pack.

10.7717/peerj.20416/supp-6 Supplemental Information 6 Between-system correlations of the peak amplitude and latency of the N200 ERP at electrode sites of interest (Fz, Cz) for each pack.

10.7717/peerj.20416/supp-7 Supplemental Information 7 Between-system correlations of the peak amplitude and latency of the P300 ERP at electrode sites of interest (Fz, Cz) for each pack.

10.7717/peerj.20416/supp-8 Supplemental Information 8 Between-system correlations of the area under the curve (AUC) and latency of the MMN ERP at electrode sites of interest (Fz, Cz) for each pack.

10.7717/peerj.20416/supp-9 Supplemental Information 9 Distribution of the SME for N170 amplitude and latency, MMN latency, compared to the distribution of the SME ERP CORE dataset at the electrode sites of interest.

## Additional Information and Declarations

### Data Availability

The following information was supplied regarding data availability:

The data is available at Zenodo: Epinat-Duclos, J. (2025). (In review) Evaluating portable EEG: A comparison between two wireless systems (EPOC Flex and LiveAmp) and the wired BrainAmp system [Data set]. Zenodo. https://doi.org/10.5281/zenodo.15698441.
