# Supplementary material for: Evaluating portable EEG: a comparison between two wireless systems (EPOC Flex and LiveAmp) and the wired BrainAmp system
Source: PeerJ. 2026 Jan 5;14:e20416. doi: 10.7717/peerj.20416 (PMC12782033; doi:10.7717/peerj.20416)
Supplement: Supplemental Information 1 [file peerj-14-20416-s001.docx]

**Supplementary material, Table 1:**

**Mean bootstrapped standard measurement error (bSME) over all participants for latency and amplitude measures for each system, waveform and electrode site in pack-100b.**

| Waveform | EEG System | Site | Peak latency bSME, ms (SE) | P2P amplitude bSME, µV (SE) |
| --- | --- | --- | --- | --- |
| N170 | EM | P7 | 8.63 (4.46) | 1.04 (0.65) |
|  |  | P8 | 9.26 (3.40) | 1.11 (1.04) |
|  | LA | P7 | 6.07 (5.49) | 0.68 (0.33) |
|  |  | P8 | 2.83 (2.23) | 0.80 (0.38) |
|  | BA | P7 | 4.63 (3.64) | 0.97 (0.36) |
|  |  | P8 | 2.76 (2.85) | 1.09 (0.46) |
|  |  |  |  | Peak amplitude bSME, µV (SE) |
| N200 | EM | Fz | 10.44 (7.42) | 2.03 (1.67) |
|  |  | Cz | 10.81 (6.93) | 1.73 (1.33) |
|  |  | Pz | 13.38 (6.01) | 1.84 (1.00) |
|  | LA | Fz | 12.31 (7.77) | 0.77 (0.33) |
|  |  | Cz | 13.95 (10.74) | 0.65 (0.21) |
|  |  | Pz | 14.47 (8.31) | 0.70 (0.24) |
|  | BA | Fz | 10.58 (9.51) | 0.87 (0.19) |
|  |  | Cz | 11.55 (11.38) | 0.86 (0.18) |
|  |  | Pz | 13.19 (7.05) | 0.91 (0.25) |
| P300 | EM | Fz | 21.76 (10.29) | 2.32 (1.89) |
|  |  | Cz | 21.70 (13.04) | 1.99 (1.48) |
|  |  | Pz | 25.46 (12.72) | 2.15 (1.28) |
|  | LA | Fz | 19.15 (11.96) | 0.85 (0.35) |
|  |  | Cz | 19.39 (13.47) | 0.72 (0.22) |
|  |  | Pz | 25.78 (17.35) | 0.75 (0.26) |
|  | BA | Fz | 17.11 (13.17) | 0.97 (0.23) |
|  |  | Cz | 15.71 (10.80) | 0.93 (0.17) |
|  |  | Pz | 22.61 (18.33) | 0.96 (0.28) |
|  |  |  |  | AUC bSME, nV.s (SE) |
| MMN | EM | Fz | 30.77 (15.62) | 42.37 (22.52) |
|  |  | Cz | 34.71 (15.24) | 37.52 (24.29) |
|  |  | Pz | 41.66 (13.55) | 38.90 (17.71) |
|  | LA | Fz | 26.39 (8.93) | 21.10 (6.85) |
|  |  | Cz | 36.52 (10.28) | 18.52 (5.68) |
|  |  | Pz | 43.44 (16.60) | 20.53 (6.53) |
|  | BA | Fz | 29.35 (12.99) | 25.68 (8.51) |
|  |  | Cz | 31.92 (13.21) | 24.98 (7.05) |
|  |  | Pz | 39.79 (13.22) | 26.28 (7.22) |
