# Supplementary material for: Evaluating portable EEG: a comparison between two wireless systems (EPOC Flex and LiveAmp) and the wired BrainAmp system
Source: PeerJ. 2026 Jan 5;14:e20416. doi: 10.7717/peerj.20416 (PMC12782033; doi:10.7717/peerj.20416)
Supplement: Supplemental Information 2 — * p ≤ 0.05, ** p ≤ 0.01, *** p ≤ 0.001. [file peerj-14-20416-s002.docx]

**Supplementary material, Table 2:**

**Statistical comparisons of the SME values to those obtained with ERP CORE data.** * *p* ≤ .05, ** *p* ≤ .01, *** *p* ≤ .001.

| ERP | Measure | Electrode | Electrode in ERP CORE | System | Mann-Whitney | Mean SME value (sd) | |
| --- | --- | --- | --- | --- | --- | --- | --- |
|  |  |  |  |  |  | Study | ERP CORE |
| N170 | Amplitude | P8 | PO8 | EM | *U* = 207, *p* = .005** | 1.11 (1.04) | 1.32 (0.41) µV |
|  |  |  |  | LA | *U* = 102, *p* < .001*** | 0.80 (0.38) |  |
|  |  |  |  | BA | *U* = 236, *p* = .020* | 1.09 (0.46) |  |
|  | Latency | P8 | PO8 | EM | *U =* 633*, p* < .001*** | 9.26 (3.40) | 4.83 (3.43) ms |
|  |  |  |  | LA | *U =* 247*, p =* .032* | 2.83 (2.23) |  |
|  |  |  |  | BA | *U =* 210*, p =* .006** | 2.76 (2.85) |  |
| MMN | Latency | Fz | FCz | EM | *U =* 607*, p* < .001*** | 30.8 (15.6) | 13.24 (6.00) ms |
|  |  |  |  | LA | *U =* 648*, p* < .001*** | 26.4 (8.93) |  |
|  |  |  |  | BA | *U =* 609*, p* < .001*** | 29.3 (13.0) |  |
|  | Latency | Cz | FCz | EM | *U =* 663*, p* < .001*** | 26.3 (15.2) | 13.24 (6.00) ms |
|  |  |  |  | LA | *U =* 696*, p* < .001*** | 36.5 (10.3) |  |
|  |  |  |  | BA | *U =* 666*, p* < .001*** | 31.9 (13.2) |  |
