# Supplementary figures and images for: Evaluating portable EEG: a comparison between two wireless systems (EPOC Flex and LiveAmp) and the wired BrainAmp system
Source: PeerJ. 2026 Jan 5;14:e20416. doi: 10.7717/peerj.20416 (PMC12782033; doi:10.7717/peerj.20416)

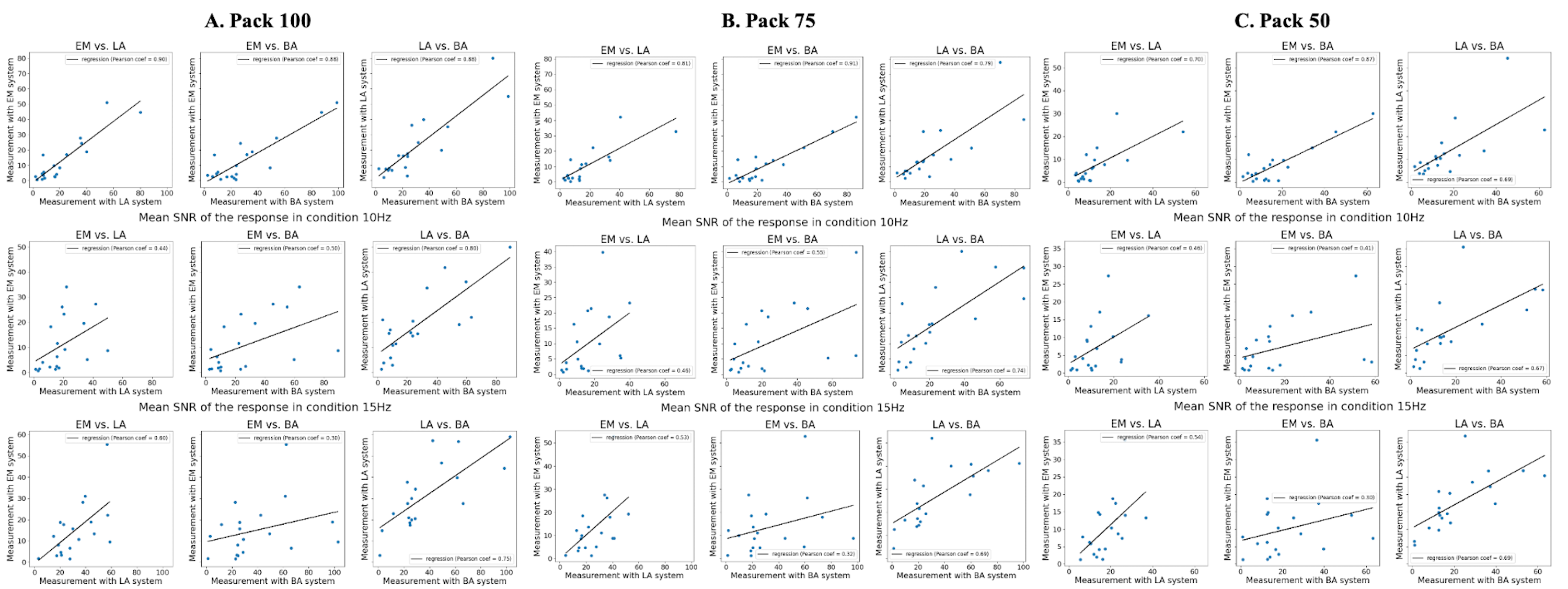

Supplement: Supplemental Information 3 [file peerj-14-20416-s003.png]

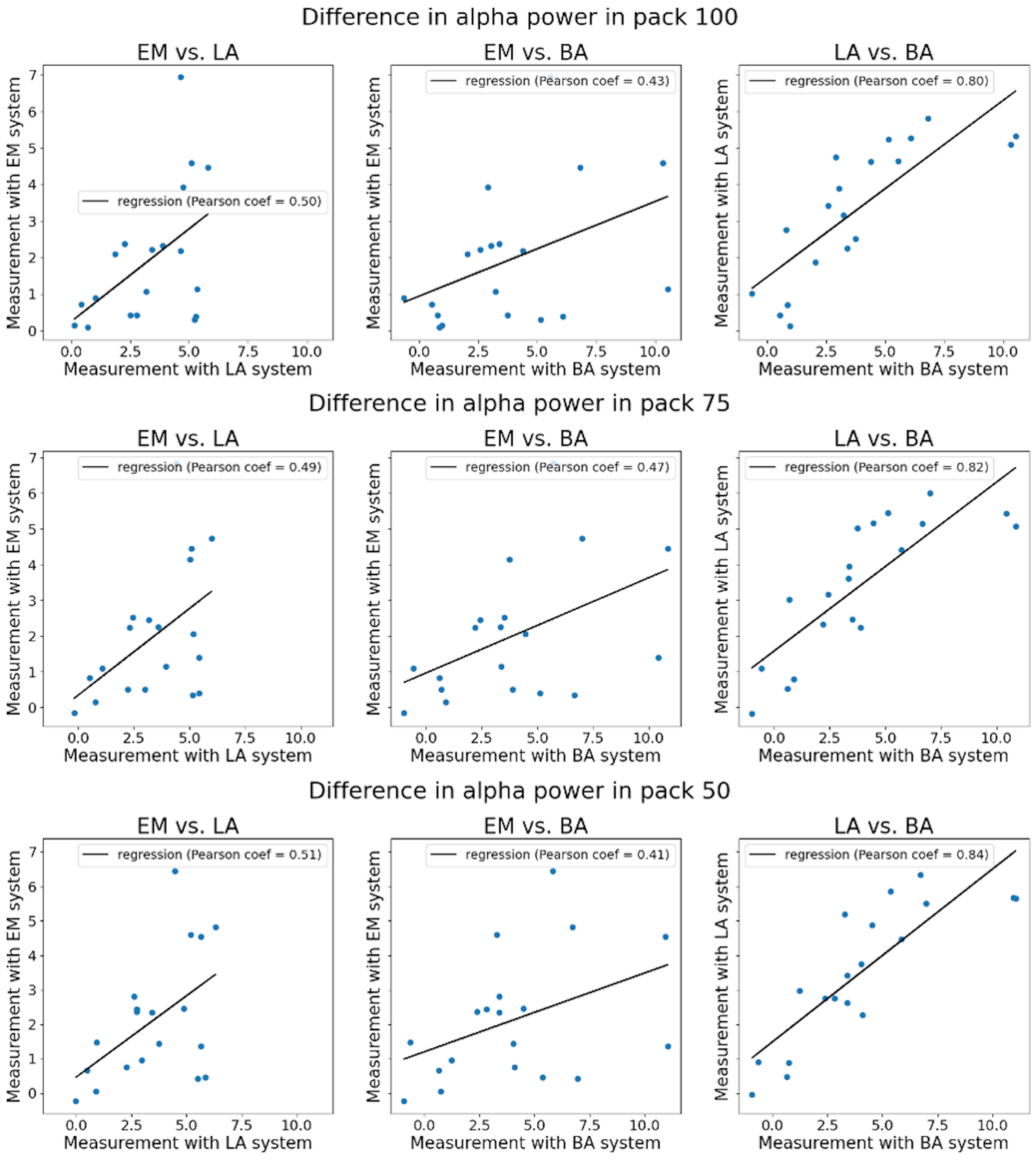

Supplement: Supplemental Information 4 [file peerj-14-20416-s004.png]

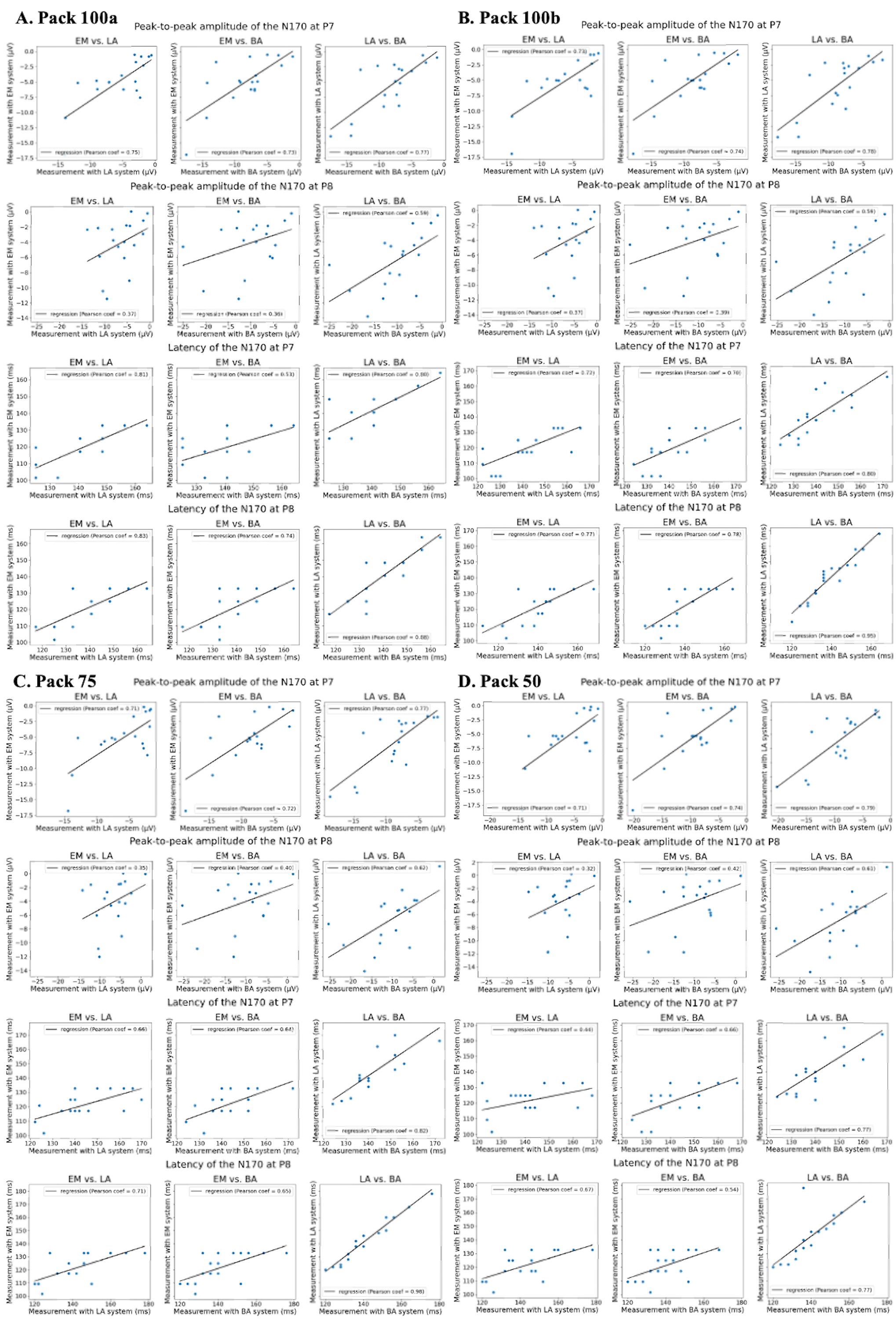

Supplement: Supplemental Information 5 [file peerj-14-20416-s005.png]

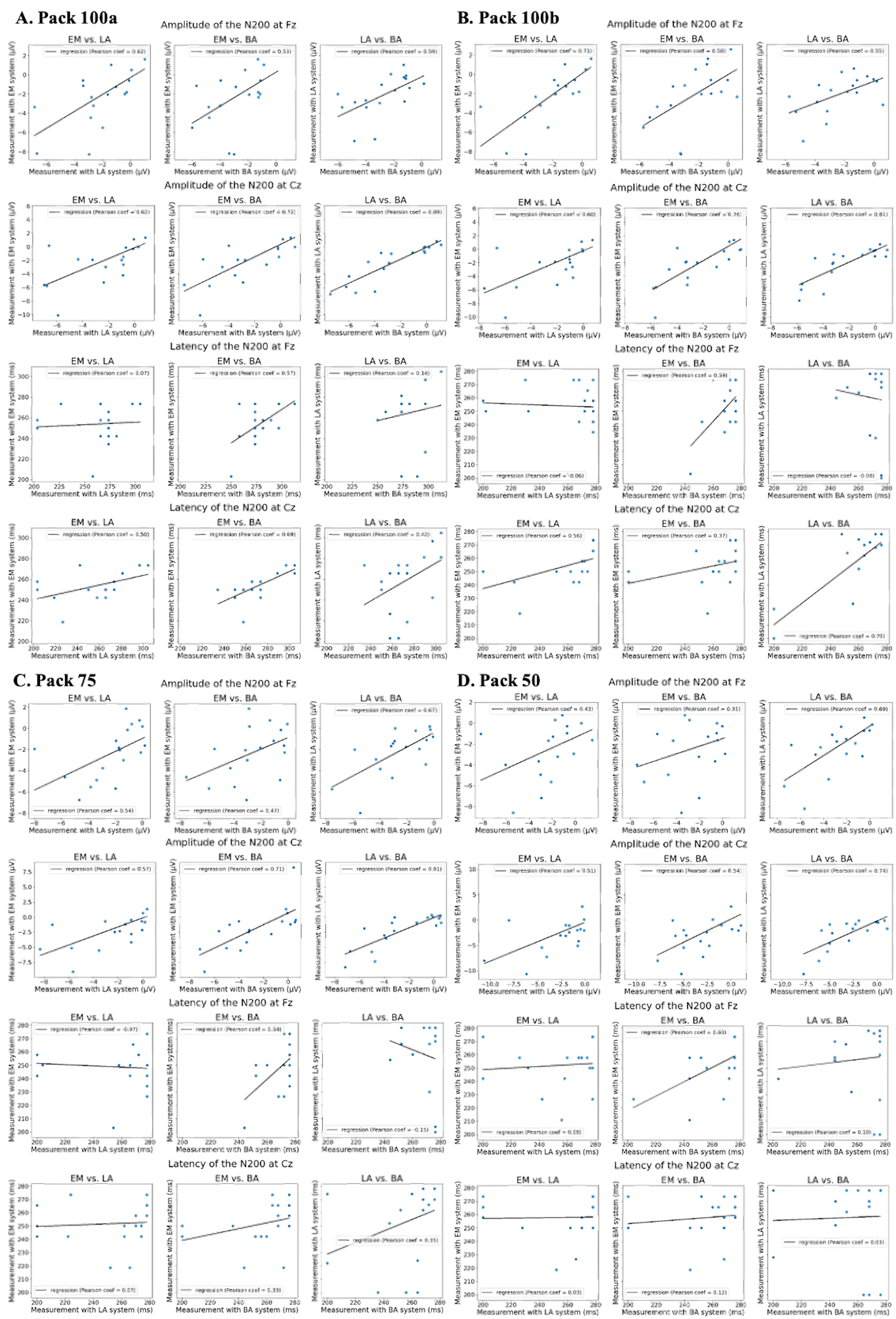

Supplement: Supplemental Information 6 [file peerj-14-20416-s006.png]

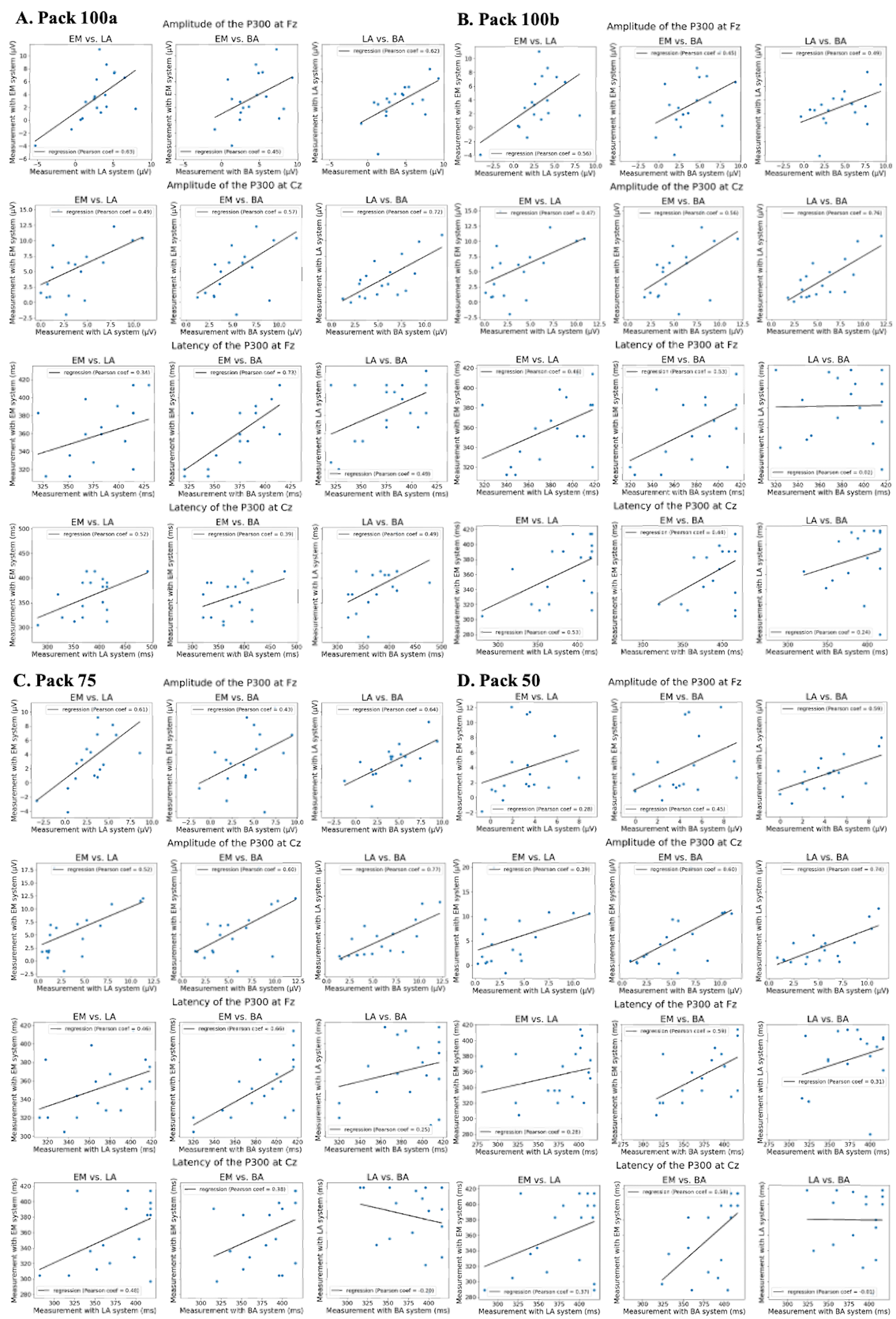

Supplement: Supplemental Information 7 [file peerj-14-20416-s007.png]

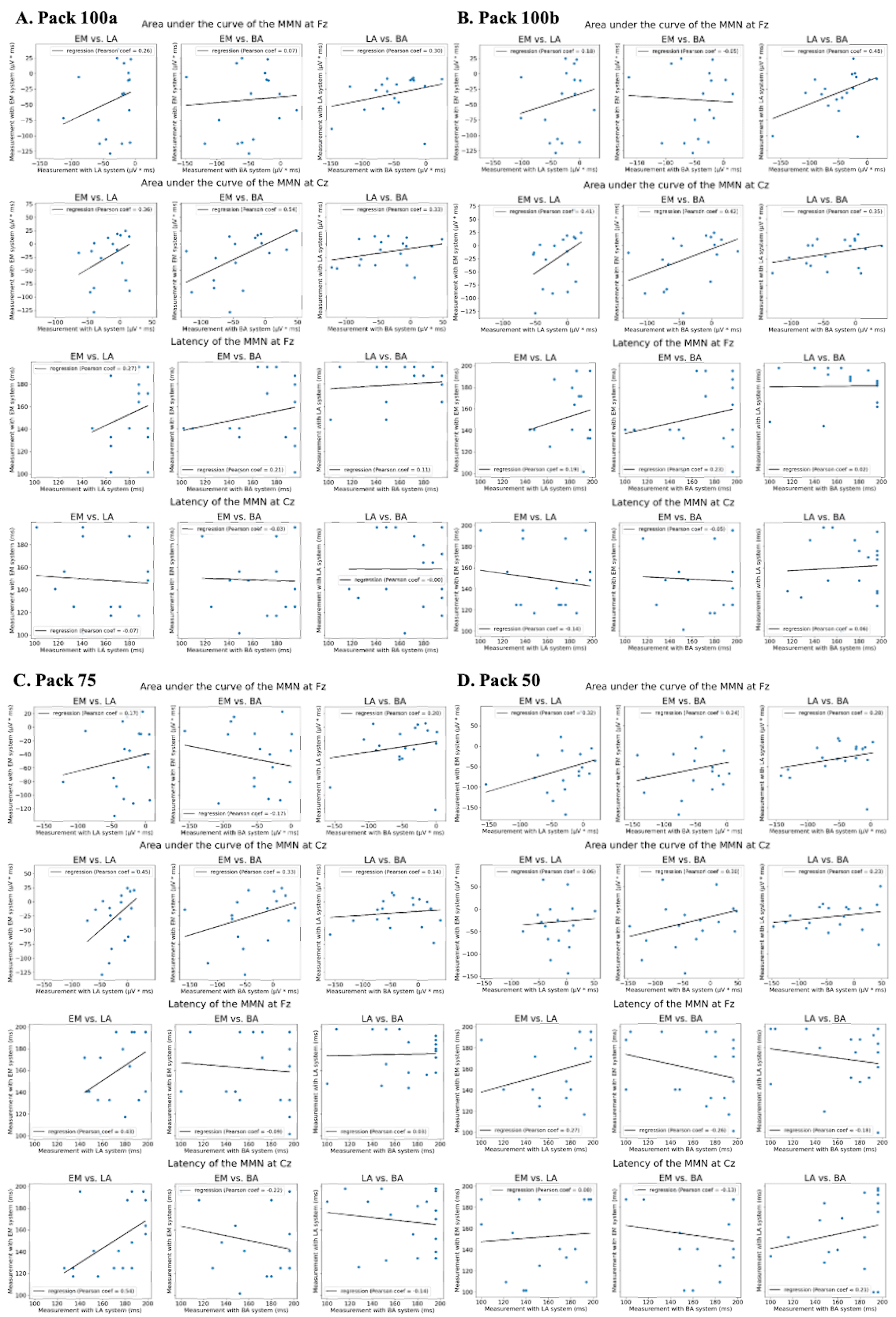

Supplement: Supplemental Information 8 [file peerj-14-20416-s008.png]

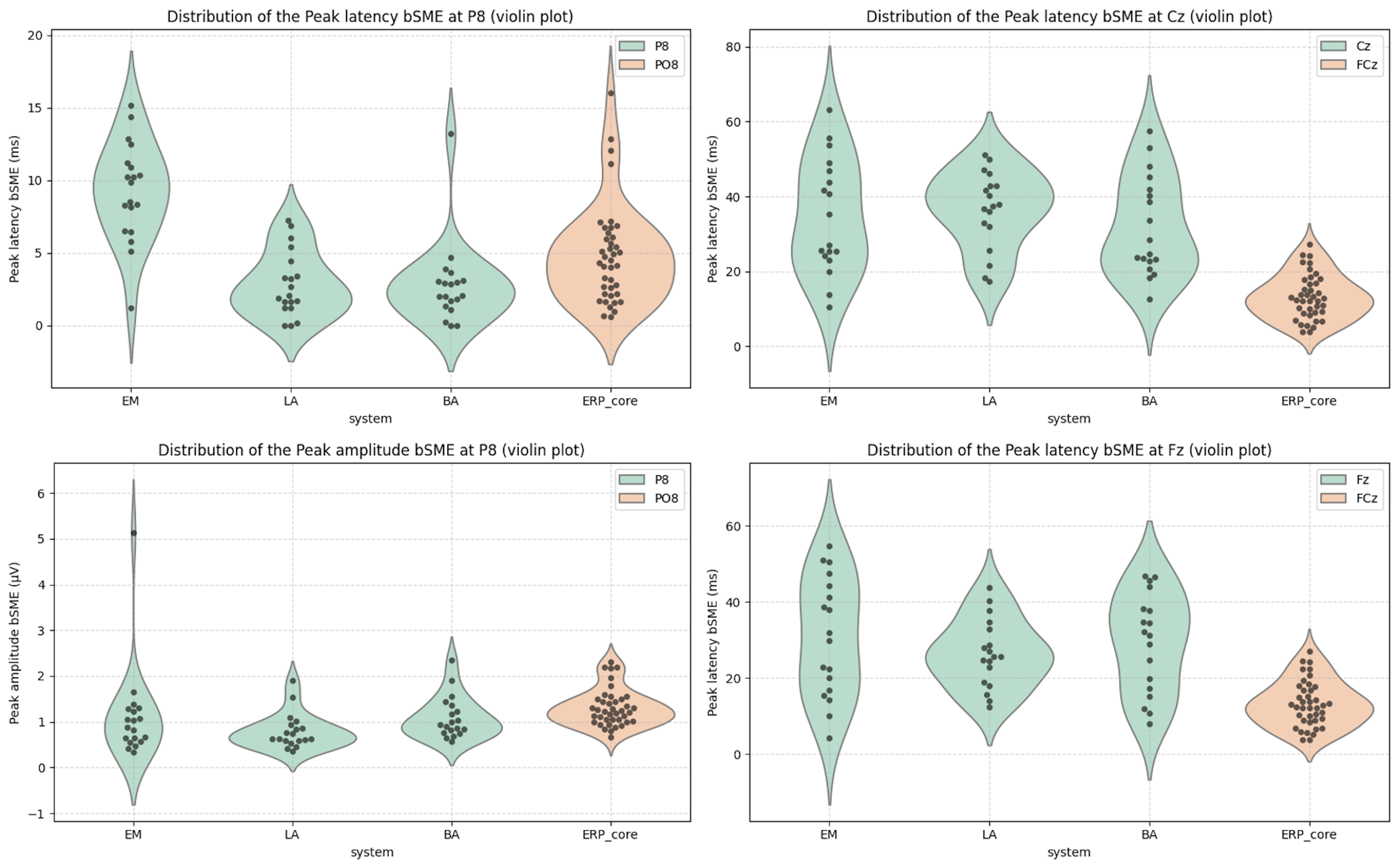

Supplement: Supplemental Information 9 [file peerj-14-20416-s009.png]
